# VCP regulates early tau seed amplification via specific cofactors

**DOI:** 10.1186/s13024-024-00783-z

**Published:** 2025-01-07

**Authors:** Sushobhna Batra, Jaime Vaquer-Alicea, Clarissa Valdez, Skyler P. Taylor, Victor A. Manon, Anthony R. Vega, Omar M. Kashmer, Sourav Kolay, Andrew Lemoff, Nigel J. Cairns, Charles L. White, Marc I. Diamond

**Affiliations:** 1 Center for Alzheimer’s and Neurodegenerative Diseases, Peter O’Donnell Jr. Brain Institute, UT Southwestern Medical Center, 6124 Harry Hines Blvd, Dallas, TX NS8.334 United States; 2 Department of Neurology, Dallas, United States; 3 Department of Pathology, Peter O’Donnell Jr. Brain Institute, University of Texas Southwestern Medical Center, Dallas, TX United States; 4 Department of Biochemistry, University of Texas Southwestern Medical Center, Dallas, TX United States; 5 Department of Clinical and Biological Sciences, Faculty of Health and Life Sciences, University of Exeter, Exeter, UK

**Keywords:** Tau, APEX2, VCP, p97, Cofactors, Disaggregase, Seeding

## Abstract

**Background:**

Neurodegenerative tauopathies may progress based on seeding by pathological tau assemblies, whereby an aggregate is released from one cell, gains entry to an adjacent or connected cell, and serves as a specific template for its own replication in the cytoplasm. Seeding into the complex cytoplasmic milieu happens within hours, implying the existence of unknown factors that regulate this process.

**Methods:**

We used proximity labeling to identify proteins that control seed amplification within 5 h of seed exposure. We fused split-APEX2 to the C-terminus of tau repeat domain (RD) to reconstitute peroxidase activity 5 h after seeded intracellular tau aggregation. Valosin containing protein (VCP/p97) was the top hit. VCP harbors dominant mutations that underlie two neurodegenerative diseases, multisystem proteinopathy and vacuolar tauopathy, but its mechanistic role is unclear. We used immortalized cells and human neurons to study the effects of VCP on tau seeding. We exposed cells to fibrils or brain homogenates in cell culture media and measured effects on uptake and induction of intracellular tau aggregation following various genetic and pharmacological manipulations of VCP.

**Results:**

VCP knockdown reduced tau seeding. Chemical inhibitors had opposing effects on seeding in HEK293T tau biosensor cells and human neurons: ML-240 increased seeding efficiency, whereas NMS-873 decreased it. The inhibitors only functioned when administered within 8 h of seed exposure, indicating a role for VCP early in seed processing. We screened 30 VCP co-factors in HEK293T biosensor cells by genetic knockout or knockdown. Reduction of ATXN3, NSFL1C, UBE4B, NGLY1, and OTUB1 decreased tau seeding, as did NPLOC4, which also uniquely increased soluble tau levels. By contrast, reduction of FAF2 increased tau seeding.

**Conclusions:**

Divergent effects on tau seeding of chemical inhibitors and cofactor reduction indicate that VCP regulates this process. This is consistent with a cytoplasmic processing complex centered on VCP that directs seeds acutely towards degradation vs. amplification.

**Supplementary Information:**

The online version contains supplementary material available at 10.1186/s13024-024-00783-z.

## Introduction

Neurodegenerative tauopathies include Alzheimer’s and related disorders that are caused by intracellular accumulation of pathological tau assemblies [1]. In each disease, pathology progresses predictably, possibly via connected neural networks [2, 3, 4, 5]. Experimental and observational evidence suggests that this occurs by release of tau aggregates, followed by their entry into a second order cell. The assembly then serves as a template for its own replication, a process termed “seeding”, which is easily replicated in simple cell models, cultured neurons, and mouse brain [6, 7, 8, 9, 10, 11].

Amplification of tau assemblies in cells can occur within hours, and in certain cases will faithfully reproduce specific assembly structures [9]. Interestingly, no seed amplification assay in vitro has yet achieved the fidelity of structural replication that occurs in cells, consistent with a role for co-factors. We originally found that tau aggregates bind heparan sulfate proteoglycans (HSPGs) on the cell surface and are taken up via macropinocytosis [12]. Most tau then traffics to the endolysosomal system where it is degraded by lysosomal proteases [13]. By contrast, a small fraction of seeding activity steadily enters the cytoplasm with clearance by the proteasome [13]. Seed amplification occurs widely throughout the cytoplasm and is not necessarily associated with the original aggregates that have been taken up [13]. These observations, and others, have led us to speculate that tau seeding is regulated by an intracellular “machinery” that brings an assembly into contact with tau monomer for amplification. Several proteomics screens from our lab and others have identified proteins associated with pre-existing intracellular tau aggregates [14, 15, 16, 17, 18], including valosin containing protein (VCP/p97) [17, 18], but we still do not understand the factors involved early in seed amplification, and conflicting reports suggest that VCP could increase [18] or decrease [19, 20] seeding by exogenous amyloids. In this study, we used proximity labeling 5 h after aggregate exposure to identify VCP as a top hit, and have characterized its regulatory role at the earliest stages of tau seed amplification.

## Results

### Proximity labeling of nascent tau aggregates identifies VCP

To identify proteins nearby tau as it initiated aggregation, we exploited split-APEX2 (sAPEX2), which renders the enzyme inactive until holoenzyme reconstitution [21]. We fused tau repeat domain (RD) containing the disease-associated P301S mutation to APEX2 fragments (AP: aa 1–200; EX: aa 201–250) each followed by an IRES sequence linked to either blue fluorescent protein or mCherry to confirm expression of both constructs. As a negative control, we used tau containing two proline substitutions (I277P / I308P) that prevented formation of beta-sheet structures [22, 23]. We also used tau RD wild type (WT) to compare to P301S and dual proline mutants. Unfused sAPEX2 expression alone controlled for background enrichment of any non-specific proteins.

**Fig. 1:**
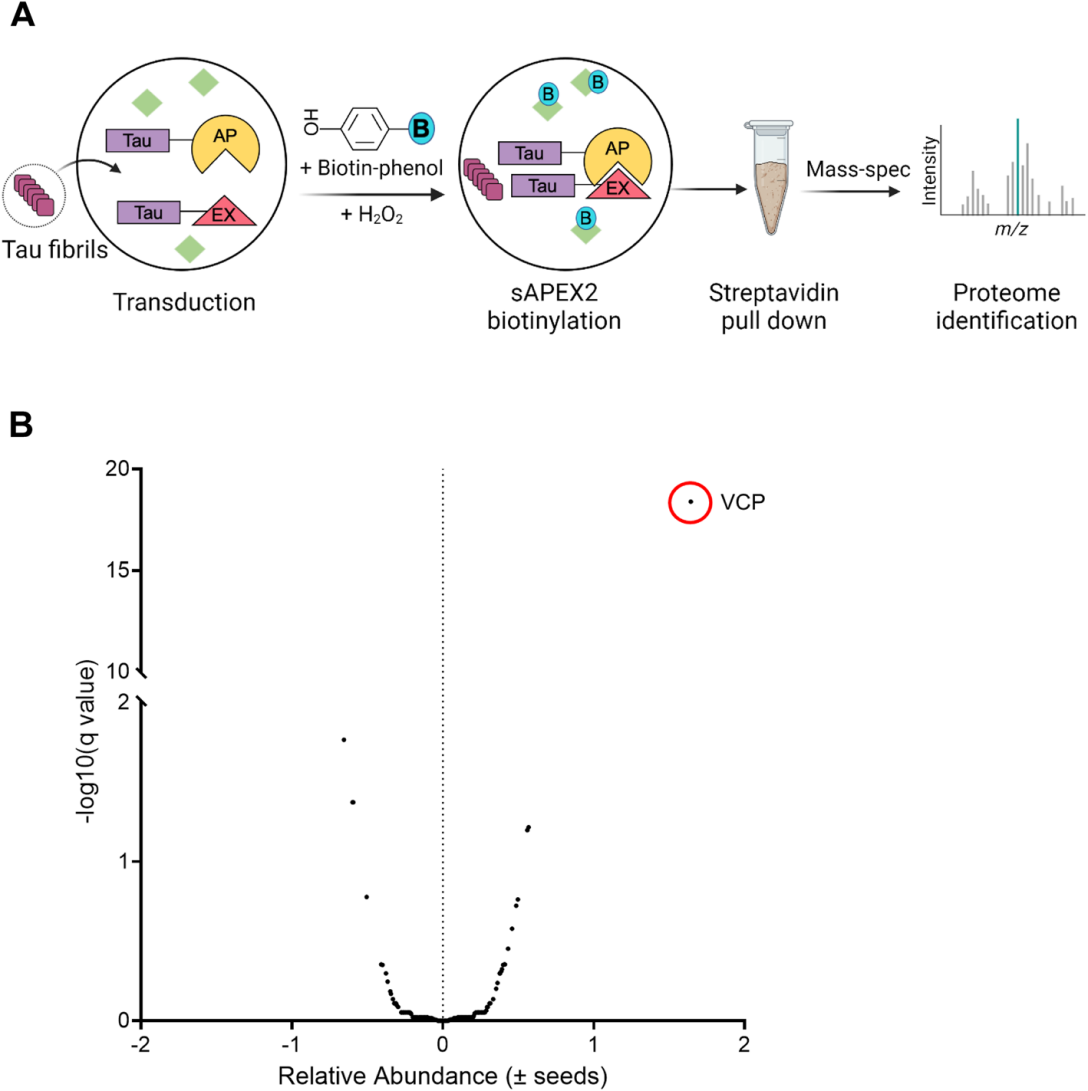
VCP identified by proximity labeling from tau aggregation. **A** Schematic of the TMT-MS study performed for proteomics. **B** VCP was the most significant hit enriched in the early tau aggregation proteome. Unpaired t- test, P value < 0.000001

We induced tau RD-AP/EX aggregation by Lipofectamine-mediated transduction of cells with full-length (FL), WT tau fibrils. The earliest detectable biotinylation occurred 5 h after induction (Supplemental Fig. 1A). Thus, after transduction of cells with tau fibrils, we waited 5 h before treating with biotin-phenol (BP) and H_2_O_2_. We then lysed the cells and used streptavidin beads to purify biotinylated proteins, which we identified using tandem mass-tag mass spectrometry (TMT-MS), pooling data from three independent experiments (Fig. 1A). VCP/p97 was the most significantly enriched hit (Fig. 1B; Supplemental Table 1).

VCP (known as Cdc48 in yeast and Ter94 in fruit flies) is a AAA + ATPase with two ATPase domains, D1 and D2. Its N-terminus binds specific cofactor/adaptor proteins that govern its diverse cellular activities [24, 25]. A dominant mutation in VCP causes vacuolar tauopathy (VT), a neurodegenerative syndrome [19], and other mutations cause multisystem proteinopathy (MSP), with protein aggregation and degeneration in brain, bone, and muscle [26, 27]. Recently, work from our lab and an independent collaboration with the Hipp and Hartl laboratories identified VCP associated with insoluble tau aggregates in cells that stably propagate inclusions [17, 18]. It has been unclear how VCP regulates intracellular seeding, given reports that it increases [18] or decreases [19, 20] it.

### VCP differentially regulates tau seeding

**Fig. 2:**
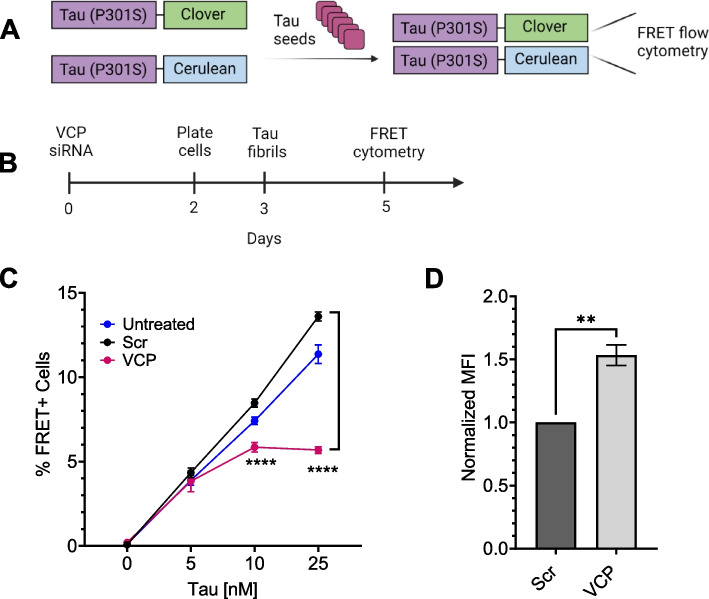
Reduction of VCP inhibits tau seeding. **A** The tau biosensor seeding assay is based on exposure of cells to exogenous tau seeds. This triggers intracellular aggregation of tau RD (P301S)-clover/cerulean that is detected by FRET. **B** Timeline of siRNA treatment for VCP knockdown (KD) in biosensors. **C** KD of VCP reduced tau seeding. Error bars represent S.D. Representative graph from *n* = 3 independent experiments, with each data point derived from technical triplicate. *P* value **** < 0.0001; One-Way ANOVA with a 95% confidence interval. **D** VCP KD increased uptake of tau fibrils labeled with AF-647, measured by flow cytometry. Error bars represent S.E.M (*n* = 3). P value ** 0.0029; Unpaired t-test with a 95% confidence interval

To quantify tau aggregation, we used v2L biosensor cells that overexpress tau RD (P301S) tagged with mClover3 and mCerulean3 (Fig. 2A) [8]. In contrast to the original APEX2 screen, for all subsequent experiments, we added recombinant tau fibrils to the media in the absence of a transfection reagent to enable HSPG-mediated macropinocytosis [7, 12, 28] and cytoplasmic seeding [6, 29, 13], which we quantified using FRET flow cytometry [7]. We genetically and pharmacologically modulated VCP activity in the biosensors to test its effects on tau seeding. Knockout (KO) of VCP is lethal, so we used siRNA-mediated knockdown (KD) (Fig. 2B), verified by western blot on cells treated with siRNA for 48 h (Supplemental Fig. 2A). VCP KD reduced tau seeding (Fig. 2C). We also observed increased basal fluorescence of the biosensor cells by microscopy (Supplemental Fig. 2B) and flow cytometry (Supplemental Fig. 2C,D). This was consistent with VCP-mediated degradation of tau monomer and made the reduction of overall seeding efficiency more remarkable (Fig. 2C; Supplemental Fig. 2E). Based on the FRET MFI values of the aggregates, we noted that the induced inclusions were larger and brighter, if less frequent (Supplemental Fig. 2F).

VCP can affect vesicular trafficking [30], so to rule out reduction of tau uptake as a cause of diminished seeding, we treated the biosensors with AF-647 (Alexa fluor-647) labeled recombinant tau fibrils for 4 h, followed by trypsin digestion to degrade extracellular fibrils. We measured uptake via flow cytometry as per standard protocols [12, 28]. VCP KD slightly increased tau uptake (Fig. 2D), ruling out diminished endocytosis as the cause of the reduced seeding.

**Fig. 3:**
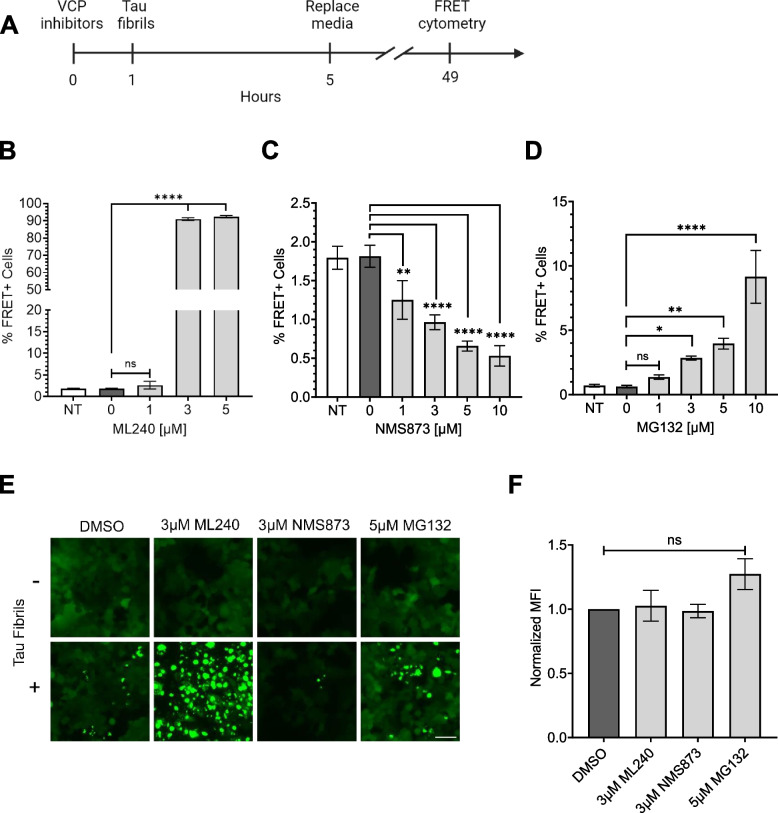
Acute exposure of inhibitors differentially impacts tau aggregation. **A** Timeline of experiment: 1 h exposure of tau biosensor cells to inhibitors, followed by 4 h of 25 nM tau before washout. **B** ML-240 increased tau seeding. *P* values: ns = 0.4353, **** < 0.0001 (**C**) NMS-873 dose-dependently decreased tau seeding**.** *P* values: ** 0.0030, **** < 0.0001 (**D**) Proteasome inhibitor, MG132, increased tau seeding. P values: ns = 0.8492, * 0.0415, ** 0.0024, **** < 0.0001. Error bars represent S.D. Representative data of *n* = 3 independent experiments, with each data point derived from technical triplicate. **E** Fluorescence microscopy confirmed the effects of VCP and proteasome inhibition on tau seeding. Scale bar = 50 μm. **F** Compound treatment did not change tau-AF-647 uptake as measured by flow cytometry. Error bars represent S.E.M (*n* = 3). P values: ns = 0.9960, 0.9992, 0.1742, in order of bars on the graph. One-Way ANOVA with a 95% confidence interval

Chronic VCP KD reduced cell proliferation over time and induced toxicity (Supplemental Fig. 2B, C). Thus, we temporarily inhibited VCP using two distinct inhibitors, ML-240 and NMS-873 (Fig. 3A). ML-240 competitively blocks ATP binding at D2, whereas NMS-873 allosterically inhibits VCP by binding to the linker between the D1 and D2 domains [31, 32, 33]. We pre-treated the cells with inhibitors for 1 h, then incubated them with tau fibrils for 4 h, followed by washout and measurement of seeding at 48 h. We observed opposing effects. ML-240 increased tau aggregation from approximately 2% to ~ 90% (Fig. 3B, E; Supplemental Fig. 3A) and speeded aggregation kinetics (Supplemental Fig. 3B). By contrast, NMS-873, reduced tau aggregation by ~ 50% (Fig. 3C, E; Supplemental Fig. 3A). Because VCP regulates protein degradation via the proteasome [31, 34, 35], we repeated the study with MG132. This increased tau seeding from ~ 1% to ~ 10% at 48 h (Fig. 3D, E; Supplemental Fig. 3A). This agreed with our prior observation that the proteasome mediates cytoplasmic clearance of seeds [13]. None of the compounds altered tau uptake (Fig. 3F).

**Fig. 4:**
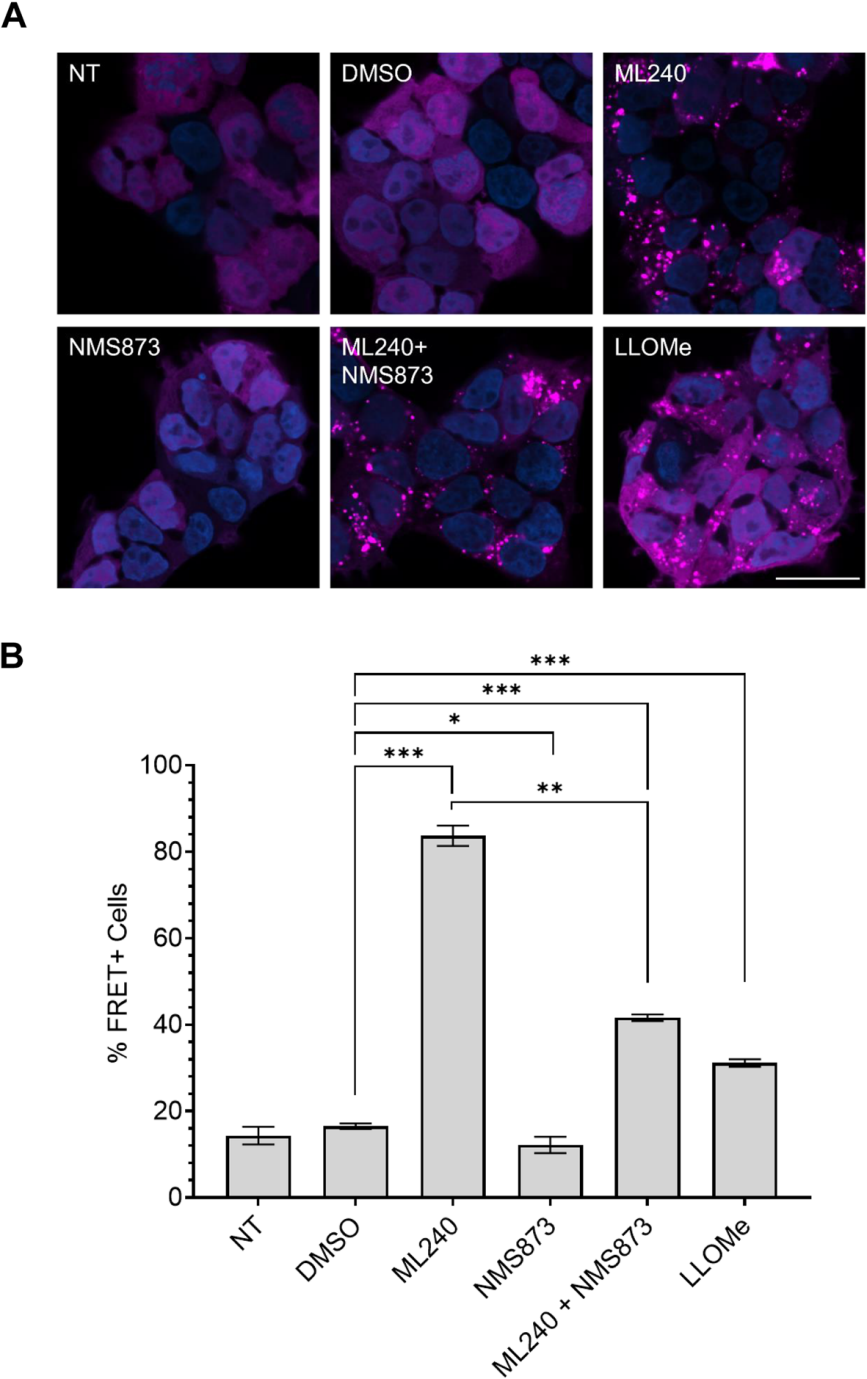
ML-240 induces Gal3 puncta formation. v2L biosensors overexpressing mRuby3-galectin3 were treated with compounds for 1 h prior to addition of tau fibrils and media replacement after 4 h. **A** Both ML-240 (3 µM) and LLOMe (1 mM) induced Gal3 puncta with no effects of NMS-873 (3 µM). Co-treatment of ML-240 and NMS-873 also induced Gal3 puncta. Representative images of *n* = 3 independent experiments taken for 5 h of compound alone incubation. Scale bar = 25 μm. **B** ML-240 increased tau seeding ~ 5x; LLOMe increased seeding by ~ 2x. NMS-873 suppressed the tau seeding enhanced by ML-240. Representative data of *n* = 3 independent experiments, with each data point derived from technical triplicate. Error bars represent S.D. *P* values: **** 0.005, * 0.0331, *** 0.0007, ** 0.0017, *** 0.0001 in order of the bars on the graph. One-Way ANOVA with a 95% confidence interval

The remarkable increase in tau seeding with ML-240 prompted us to check if its effects were mediated via vesicle rupture, thereby increasing the amount of tau seeds available in the cytoplasm for amplification. We utilized v2L biosensors overexpressing galectin-3 tagged to mRuby3 fluorophore (v2L-Gal3) to visualize Gal3 puncta formation. We also overexpressed the same construct (mRuby3-Gal3) in U2OS cells for imaging. We used L-Leucyl-L-leucine methyl ester (LLOMe) as a positive control to induce endolysosome rupture [36, 37, 38]. Brief exposure of both v2L-Gal3 and U2OS cells with ML-240 and LLOMe induced Gal3 puncta formation, indicating vesicle rupture (Fig. 4A; Supplemental Fig. 4A), however ML-240 enhanced seeding far more than LLOMe (Fig. 4B). NMS-873 did not induce Gal3 puncta (Fig. 4A; Supplemental Fig. 4A). When we co-treated cells with ML-240 and NMS-873, Gal3 puncta formed (Fig. 4A; Supplemental Fig. 4A) but seeding was relatively attenuated (Fig. 4B), indicating that NMS-873 likely inhibited a process downstream of vesicle rupture.

**Fig. 5:**
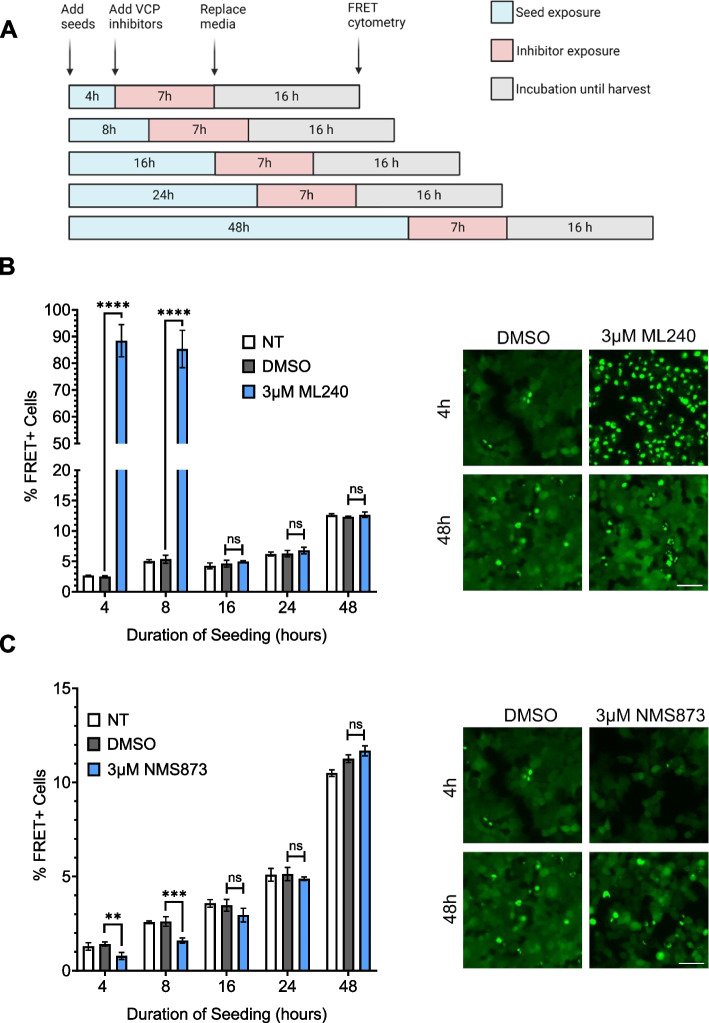
VCP inhibition impacts tau seeding early in the process.** A** Timeline of compound and tau treatments at different time points in the seeding process. **B** ML-240 increased tau aggregation ~ 16 to 25-fold, but only when administered < 8 h after seed exposure. Representative images are shown in the right panel. Scale bar = 50 μm. P values: **** < 0.0001, ns (16hr) = 0.5892, ns (24hr) = 0.4340, ns (48hr) = 0.3569. **(C) **NMS-873 decreased tau seeding by ~50%, but only when administered <8h after seed exposure. Representative images are shown in the right panel. Scale bar = 50μm. P values: ** 0.0039, *** 0.0004, ns (16hr) = 0.0788, ns (24hr) = 0.8695, ns (48hr) = 0.0547. Error bars represent S.D. Representative data of n=3 independent experiments, with each data point derived from technical triplicate. One-Way ANOVA with a 95% confidence interval

VCP regulates protein degradation, among other functions, and thus could impact seeding through clearance of aggregates over time. To resolve this issue, we added inhibitors for 7 h beginning at different time points after initial seed exposure (Fig. 5A). The inhibitors changed tau seeding only when administered within the first 8 h, and we observed no effect on seeding after that timepoint (Fig. 5B, C; Supplemental Fig. 5A). These results indicated that VCP regulates aggregation early in the seeding process.

**Fig. 6:**
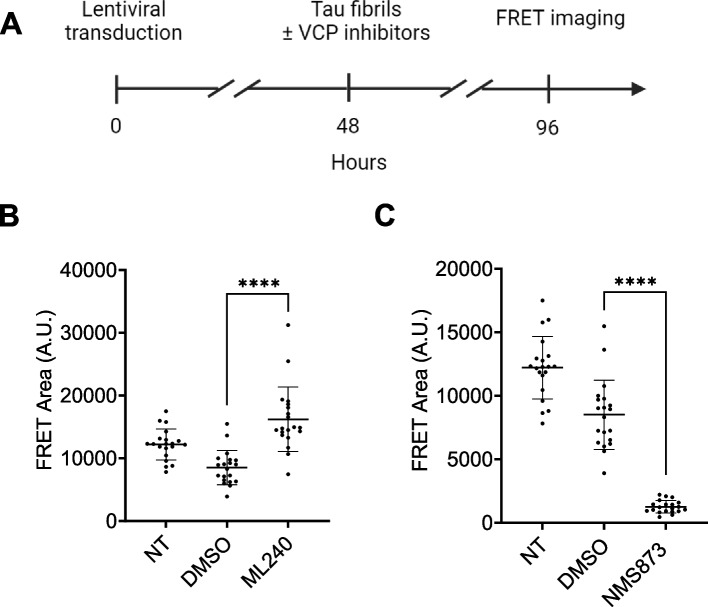
VCP inhibitors differentially impact tau seeding in human neurons. **A** Differentiated iPSC-derived human neurons were transduced with tau RD (P301S)-clover/ruby lentivirus for 48 h followed by seeding in the presence or absence of 100 nM VCP inhibitors. **B** ML-240 enhanced tau seeding in neuronal biosensors whereas **C** NMS-873 suppressed tau seeding. Error bars represent S.D. Representative data of *n* = 3 independent experiments. Each dot represents an image taken per condition, with 4 different locations captured per well, for a total of 5 wells per condition. *P* value: **** < 0.0001; Paired t-test with a 95% confidence interval

To test the effects of VCP inhibitors in a more physiologically relevant system, we measured tau seeding in iPSC-derived human neurons treated with ML-240 and NMS-873. We first transduced differentiated neurons with lentivirus to express tau RD (P301S)-Clover/Ruby biosensor proteins. After 48 h, we treated cells with ML-240 or NMS-873 at 100 nM along with tau fibrils for an additional 48 h and measured FRET (Fig. 6A). We also measured seeding in human neurons treated with tau and the inhibitors for 4 h (ML-240 at 1 μM and NMS-873 at 100 nM) prior to washout and culture for 48 h (Supplemental Fig. 6A). We quantified seeding by high content FRET microscopy (Image Xpress). ML-240 increased and NMS-873 decreased tau seeding (Fig. 6B,C; Supplemental Fig. 6B, C, D).

### ML-240 increases AD and CBD brain lysate seeding

**Fig. 7:**
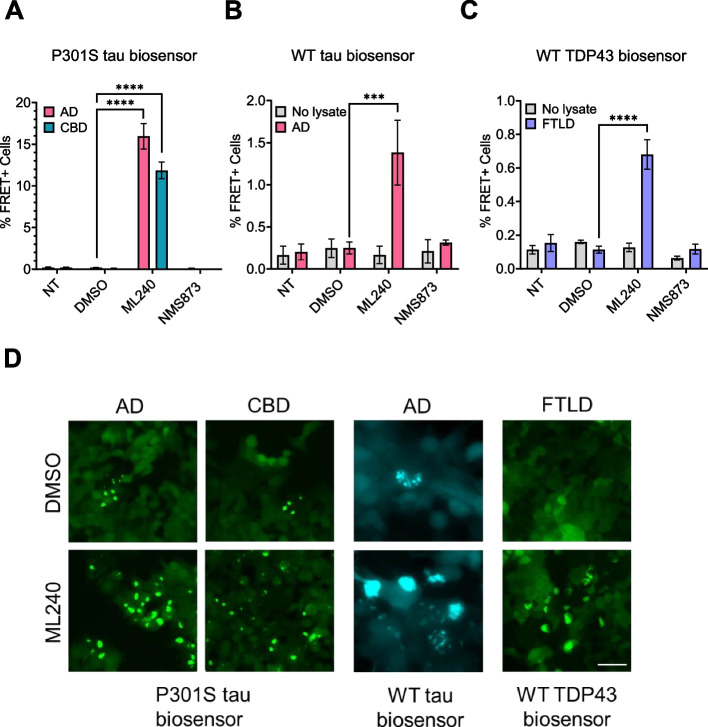
ML-240 enhances seeding by human brain lysates. **A** ML-240 increased seeding by AD and CBD brain samples onto v2L P301S tau biosensors and (**B**) by AD brain sample onto WT tau (3R/4R) biosensors. **C** ML-240 increased seeding of FTLD type A brain lysate onto TDP-43 biosensors. Error bars represent S.D. Representative data of *n* = 3 independent experiments, with each data point derived from technical triplicate. *P* value: **** < 0.0001, *** 0.0008; One-Way ANOVA with a 95% confidence interval. **D** Representative microscopy images showing effects of ML-240 on tauopathy lysates and TDP-43 seeding. Scale bar = 50 μm

To test the effects of VCP inhibitors on a physiological tau seed source, we tested tauopathy brain lysates from AD (Alzheimer’s disease) and CBD (corticobasal degeneration) patients. We treated biosensor cells with ML-240 and NMS-873 as described above, after which they were exposed to AD and CBD patient brain lysates without a transduction reagent. ML-240 increased AD and CBD seeding by ~ 10x (Fig. 7A, D). Since the lysates induced very low seeding in the absence of a transduction reagent, we could not test the effects of NMS-873.

We also tested the effects of VCP inhibitors on WT tau biosensors, HEK293T cells overexpressing the 3R isoform fragment of WT tau aa 246–408 tagged to mCerulean3, and the 4R version of the same sequence tagged to mRuby3. ML-240 enhanced seeding by AD lysate in these cells (Fig. 7B, D). The effect of NMS-873 could not be tested due to the low seeding efficiency of AD lysate on these cells.

### ML-240 increases TDP-43 brain lysate seeding

To test whether VCP regulated seeding by another amyloid, we evaluated the effects of the inhibitors on biosensors expressing WT TDP-43 (aa 275–414) fused to mClover3 or mRuby3 fluorescent proteins. ML-240 enhanced FTLD-TDP Type A brain lysate seeding on TDP-43 biosensors (Fig. 7C, D). Since the lysate induced no seeding in the absence of a transduction reagent, we could not test the effects of NMS-873.

### VCP co-factors regulate tau aggregation

Multiple co-factors generate specificity for VCP’s myriad cellular functions [24, 39, 40]. To identify those which regulated seeding in v2L tau biosensors, we individually knocked out or reduced the expression of 30 cofactors known to be expressed in HEK293T cells [41] (Table 1).

**Table 1:** List of VCP cofactors and their proposed functions

Cofactor	Function
AMFR/GP78	ERAD
ANKZF1	Cellular response to hydrogen peroxide, ERAD
ASPSCR1/ UBXD9	VCP hexamer disassembly
ATXN3	Deubiquitinase; ERAD
DERL1	ERAD
DERL2	ERAD
FAF1/UBXD12	Apoptosis, autophagy
FAF2/UBXD8	ERAD, lipid droplet turnover
NGLY1	Degradation of misfolded glycoproteins
NPLOC4/NPL4	ERAD
NSFL1C/p47	Membrane fusion
OTUB1	Cleaves branched polyubiquitin chains
PLAA	ERAD, autophagy
RPS27A	Fusion of ubiquitin and ribosomal protein S27a
SVIP	ERAD, autophagy
SYVN1	E3 ligase; ERAD
UBE4B	E3/E4 ligase; ERAD
UBXN1/SAKS1	ERAD
UBXN10/UBXD3	Tethering factor for VCP in cilium assembly
UBXN11/UBXD5	Actin cytoskeleton reorganization
UBXN2A/UBXD4	Autophagosome formation, proteasome degradation
UBXN2B/p37	Membrane fusion
UBXN4/UBXD2	ERAD
UBXN6/UBXD1	ERAD, endosome to lysosome transport, macroautophagy
UBXN7/UBXD7	HIF1α turnover
UBXN8/UBXD6	ERAD
UFD1L	ERAD
VCPIP1	Deubiquitinase, membrane fusion
VIMP	ERAD
YOD1	Deubiquitinase, macroautophagy, ERAD

**Fig. 8:**
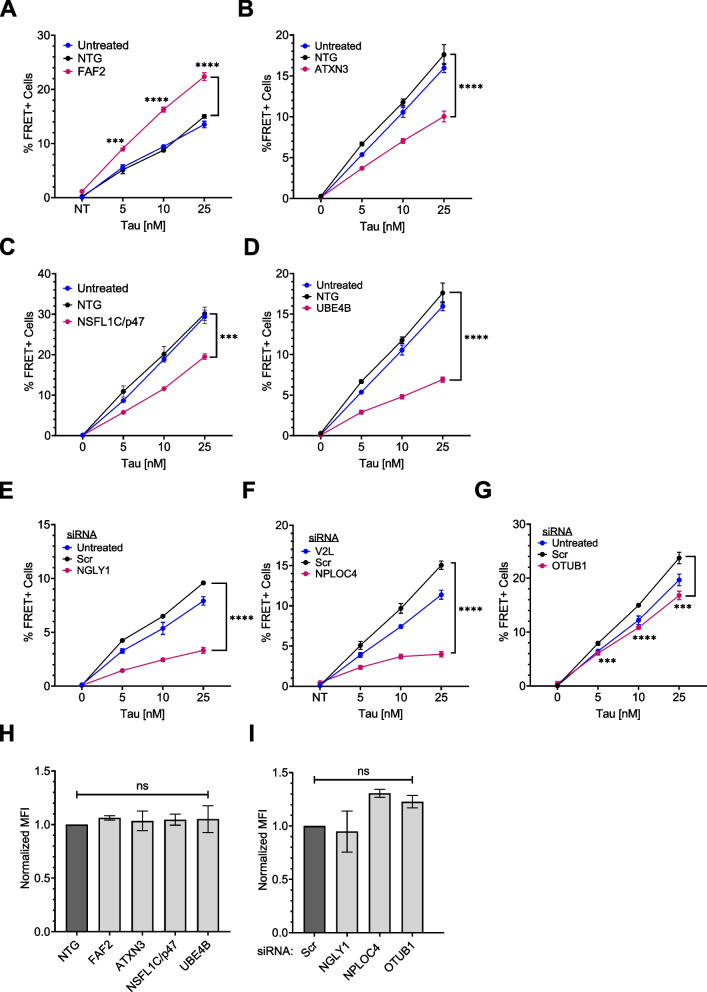
VCP cofactors differentially regulate tau seeding. VCP cofactors were either knocked out via CRISPR/Cas9 (**A-D**) or knocked down via siRNA (**E–G**) in v2L biosensors prior to exposure to increasing amounts of tau fibrils. **A** Knockout of FAF2 increased tau seeding whereas knockout of **B** ATXN3, **C** NSFL1C, and **D** UBE4B reduced tau seeding. P values: FAF2 (*** 0.0001, **** < 0.0001); ATXN3 (**** < 0.0001); NSFL1C (*** 0.0002); UBE4B (**** < 0.0001). **E** Knockdown of NGLY1, **F** NPLOC4, and **G** OTUB1, decreased tau seeding. P values: NGLY1(**** < 0.0001); NPLOC4 (**** < 0.0001); OTUB1 (*** 0.0004, **** < 0.0001, *** 0.0001). Graphs are representative of *n* = 3 independent experiments, with each data point derived from technical triplicate. Error bars represent S.D. Some error bars are too small to be visible. **H** Cofactor KO did not affect tau uptake. *P* values: ns = 0.9819, 0.9988, 0.9956, 0.9928, in order of bars on the graph. **I** Cofactor KD did not affect tau uptake. *P* values: ns = 0.9795, 0.1856, 0.3928, in order of bars on the graph. Error bars represent S.E.M (*n* = 3). One-Way ANOVA with a 95% confidence interval

For CRISPR/Cas9 KO, we used four gRNAs per gene from the Brunello library [42], compared to 4 non-targeting guides (NTG) combined as a negative control. For genes that were toxic upon KO, we used siRNA-mediated KD, with scrambled (Scr) siRNA as a negative control. KO or KD of most cofactors did not change tau seeding (Supplemental Fig. 8A). RPS27A was the only cofactor for which both KD and KO were lethal and thus we could not determine its effects on seeding. Knockout of UBXN6 increased tau seeding but the effect was most pronounced at higher tau concentrations (Supplemental Fig. 8B). KO of FAF2 alone clearly increased tau seeding (Fig. 8A), and even induced spontaneous aggregation in the biosensors in the absence of any exogenous tau fibrils (Supplemental Fig. 8C, D). In contrast, KO of the deubiquitinase ATXN3, and the E3/4 ligase UBE4B, suppressed seeded tau aggregation (Fig. 8B, D). KO of NSFL1C also reduced seeding (Fig. 8C). We confirmed each KO by western blot (Supplemental Fig. 8E). siRNA KD of three cofactors decreased seeding: NGLY1, NPLOC4, and OTUB1 (Fig. 8E, F, G). Remarkably, despite reducing seeding, KD of NPLOC4 increased tau levels in the biosensors (Supplemental Fig. 8F, G, H). NPLOC4 KD increased inclusion size, whereas NGLY1 KD reduced fluorescence and created smaller puncta (Supplemental Fig. 8F). We confirmed each effective KD by western blot (Supplemental Fig. 8I). No cofactor KO or KD changed tau uptake (Fig. 8H, I).

## Discussion

It is unknown whether a cellular regulatory machinery might control seeding by tau, especially within the initial hours of cell entry. To identify factors that participate in tau seed amplification within 5 h of exposure, we used proximity labeling to identify VCP, which has previously been genetically and biochemically linked to chronic tau aggregation [17, 18, 19], and to inhibition of α-synuclein and TDP-43 seeding [20]. Chemical manipulations of VCP up- and down- regulated tau seeding in immortalized cells and human neurons. Interestingly, these effects only manifested if inhibitors were applied within 8 h of seed exposure. We observed these effects also with brain-derived tau and TDP-43 seeds, along with another amyloid substrate, recombinant α-synuclein fibrils (Supplemental Fig. 7A, B). We identified select VCP cofactors that participate in this differential regulation, suggesting that a complex within the cell processes incoming tau seeds, either to decrease or increase their replication efficiency.

### VCP processes seeds to determine their fate

Cells can take up tau fibrils through multiple mechanisms including HSPG-mediated macropinocytosis [12, 28, 43], LRP1-facilitated endocytosis [44], and even direct translocation into the cytoplasm [45]. Our prior work defined a trafficking pathway for vesicular tau seeds that delivers them to the cytoplasm, where they are cleared by the proteasome [13]. Ordinarily, seeding efficiency from aggregates entering the cell via endocytosis is relatively low, consistent with robust clearance mechanisms [13]. However, we observed a dramatic increase in seeding efficiency for recombinant and patient-derived seeds in the presence of ML-240, a VCP inhibitor that targets the D2 ATPase. Conversely, NMS-873, an allosteric inhibitor, reduced tau seeding by ~ 50%, as did knockdown of VCP. We observed a seemingly paradoxical effect in the context of knockdown of VCP and NPLOC4: an increased tau steady state and reduced seeding. This suggests a core function of VCP in up- and down-regulating tau seeding that is linked to, but functionally independent from tau degradation.

**Fig. 9:**
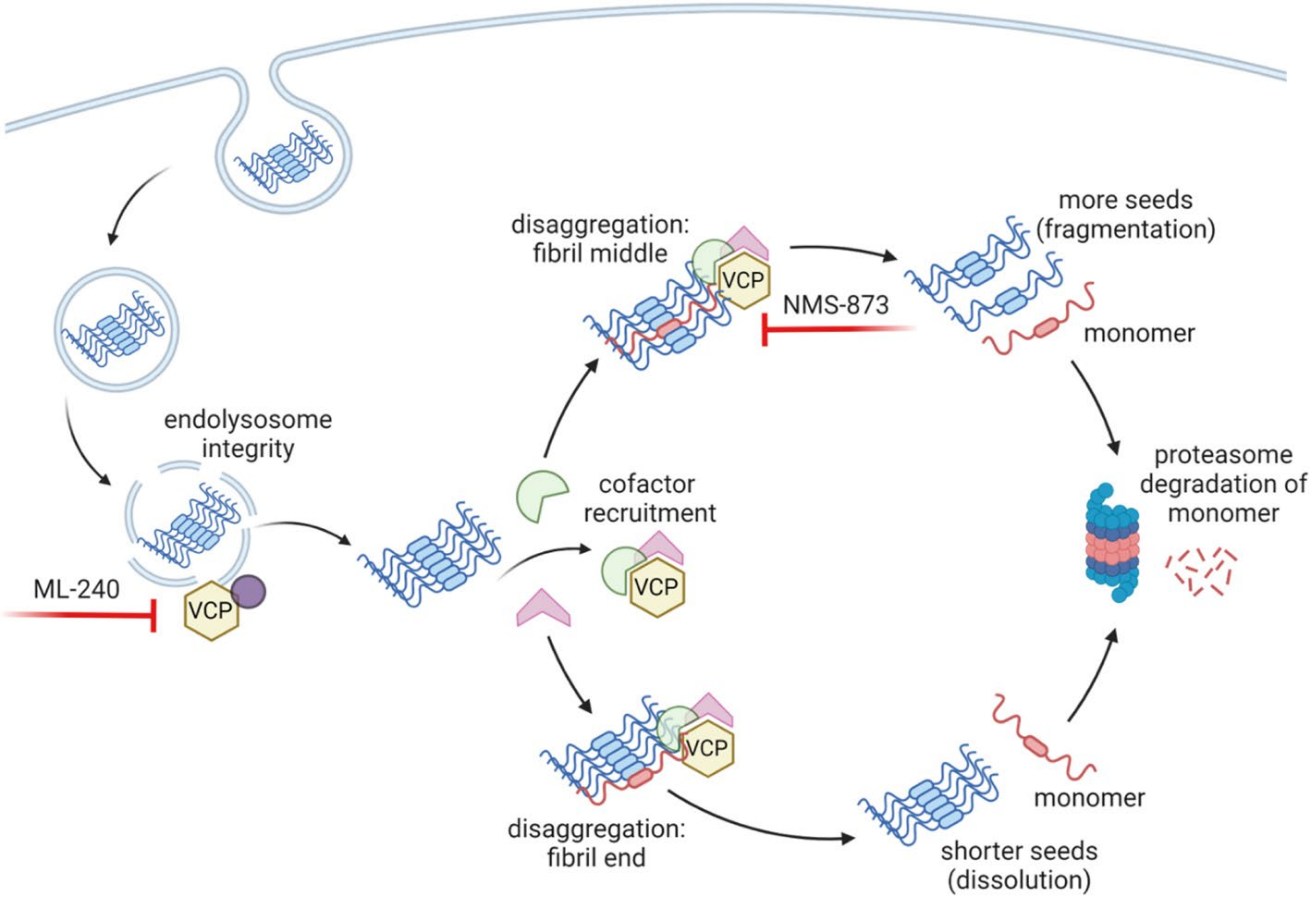
VCP regulation of tau seeding. VCP regulates endolysosomal integrity, which governs the amount of tau seeds escaping into the cytoplasm. VCP then acts on tau seeds that enter the cytoplasm to promote or inhibit either their degradation or amplification. Disaggregase activity of VCP removes monomer for degradation. This might occur at the end of filaments, which would decrease seeding, or from within, which could fragment fibrils and promote seeding. Chemical inhibitors and cofactors bias the process towards different paths

A model (Fig. 9) represents a VCP-mediated balance of forces that decrease or increase seeding, and is based largely on studies of the yeast prion disaggregase, Hsp104, which regulates prion propagation and dissolution [46, 47, 48]. Synthesizing our recent data with prior work on VCP and Hsp104, we hypothesize that VCP regulates seeding in two ways. First, by influencing membrane repair, it might affect seed escape through the endolysosomal membranes. Second, depending on where it extracts tau monomer from the amyloid assembly (middle vs. end), it could progressively reduce fibril length (to reduce seeding) or fragment fibrils (to increase seeding) (Fig. 9).

Others have proposed slightly different models for VCP’s effects on amyloids. Darwich et al. suggested that the vacuolar tauopathy mutation reduces VCP’s disaggregase activity [19], and hence clearance of existing tau aggregates; whereas Zhu et al. proposed that VCP-mediated surveillance of damaged vesicles and lysophagy suppresses α-synuclein aggregation, although this was based on cationic lipid-mediated amyloid delivery to biosensors that bypassed standard endocytosis [20]. Endolysosome rupture has been observed to facilitate enhanced tau seeding as mediated by ESCRT III machinery proteins such as CHMP6 [49], and recent work also indicates that lysosomal micro-rupture (without production of Gal3-postive puncta) allows exogenous seeds to access the cytoplasm [50]. It is now well established that VCP, along with specific cofactors, regulates the endolysosomal damage response (ELDR) pathway which is altered by VCP disease-associated mutations [51, 52, 53, 54]. Taken together with findings here and previously reported [13], it seems likely that VCP regulates early seed access to the cytoplasm based on control of endolysosomal leakage. Additionally, VCP appears to control fibril fragmentation, because NMS-873 counteracted the ML-240-mediated increase in tau seeding. VCP regulated seeding into biosensors expressing mutant and WT tau, and WT TDP-43, suggesting that the mechanisms likely apply across multiple amyloid proteins, and in sporadic disease. The model makes specific predictions that will require further testing to identify the precise mechanisms by which VCP prevents [19, 20, 55] or promotes [18] seeding by tau and other amyloid proteins.

### VCP functions early in the seeding process

Proximity labeling identified proteins adjacent to newly formed tau aggregates at the earliest possible time point and identified VCP as the single most reliable hit. The low number of identified proteins may be because a low level of APEX2 holoenzyme reconstitution restricted the labeling efficiency. We and others have clearly observed VCP association with mature aggregates [17, 18], implying additional roles in chronic protein quality control. However, the restriction of VCP’s effects on seeding to early in the process indicates its critical role in processing of tau seeds as they first enter the cytoplasm. We speculate that ML-240’s effect is most pronounced in the initial stages because it facilitates the escape of newly engulfed tau seeds into the cytoplasm for subsequent amplification. We do note conflicting reports about how seeds might exit the endolysosomal system [13, 29, 50]. We observed a similar temporal effect for NMS-873 on cytoplasmic seeding. Moreover, NMS-873 suppressed the seeding induced by ML-240, likely by preventing the fragmentation of seeds that have escaped into the cytoplasm. VCP’s role in seed amplification thus appears restricted to early stages.

### VCP cofactors dictate the fate of tau seeds

Multiple efforts have attempted unsuccessfully to target VCP with small molecules to treat cancer [56]. Our observations with ML-240 and NMS-873, which we originally predicted would have the same effect on tau seeding, highlighted the mechanistic complexity of this enzyme. Indeed, we found that specific cofactors were necessary for VCP to control seed amplification vs. destruction. Knockout of FAF2, also known as UBXD8, strongly enhanced tau seeding. It also caused spontaneous aggregation in the biosensors, which we had never before observed. FAF2 was recently reported to facilitate VCP-dependent disaggregation of stress granules [57]. It is a component of ERAD, with important roles also in lipid droplet biogenesis and maintenance [58, 59]. It is unclear whether FAF2’s effects on tau seeding arise from a complex with tau and VCP or via an indirect effect.

Genetic deletion of ATXN3 (Ataxin 3), a deubiquitinase, suppressed tau seeding. Ataxin3 is proposed to facilitate ubiquitinated substrate release from VCP by cleaving ubiquitin chains to a minimum length for proteasomal degradation [60]. However, its exact role in processing VCP substrates remains unclear. In the absence of its deubiquitinase activity in yeast, Cdc48 cannot thread substrate through the core [61]. Thus, ATXN3 KO might also prevent fragmentation of assemblies. Intriguingly, KO of an E3/4 ligase, UBE4B, also reduced tau seeding. We hypothesize that a specific ubiquitin signature on tau fibrils might be required for processing by VCP. Thus, in the absence of UBE4B, the fibrils might lack the ubiquitination required for efficient disaggregation by VCP, reducing their fragmentation and resultant seeding. However, we can’t exclude the possibility that these cofactors could alter the ubiquitin state of another substrate that secondarily regulates tau seeding. The exact role of the ubiquitin/deubiquitination pathway in tau seed processing requires further investigation.

To test whether the cofactor knockouts that increased tau seeding affected the endolysosomal pathway, as observed with ML-240, we overexpressed mRuby3-Gal3 in the FAF2 and UBXN6 KO cells. We observed no basal Gal3 puncta in these cells, either in the presence or absence of tau fibrils (Supplemental Fig. 8J). It is unknown whether identified cofactors alter tau seeding via regulation of vesicular integrity in a manner consistent with observations from the Hurley lab [50]. Taken together, our results point to a complex molecular machine, likely with cell-specific components, that determines the fate of tau seeds.

### Multiple roles of VCP in degenerative disorders

Distinct VCP mutations cause VT [19] or MSP with ubiquitinated aggregates of TDP-43 [62, 63]. MSP mutants have been proposed to be hyperactive [64, 65] whereas VT could result from VCP hypoactivity [19]. However, in vitro experiments of ATP hydrolysis in isolation likely cannot account for the complexity of VCP mutations in a cellular setting. Indeed, others suggest that they might alter cofactor binding [60, 65, 66], perhaps explaining the diverse phenotypes. Multiple reports, including ours, now indicate that VCP regulates aggregation of TDP-43, α-synuclein, and tau through diverse mechanisms including autophagy, endolysosomal degradation, and disaggregation [19, 67, 20, 18].

We must now consider VCP’s role early in the seeding process, and the specific cofactors involved in these activities. These could be conformation (strain) or amyloid-specific. Indeed, others identified UBXN6/UBXD1 as a VCP cofactor that regulates α-synuclein seeding in neurons [20]. It is possible that neurons might utilize cofactors different from those identified in HEK cells to regulate tau seeding, and this will await additional comprehensive study. Ultimately, inhibition of specific sub-functions of VCP by modulating key cofactor interactions might be the only way to therapeutically target an enzyme otherwise critical for cell viability.

## Methods

### Cell culture

HEK293T and U2OS cells were obtained from ATCC to create all cell lines. Cells were maintained in Dulbecco’s DMEM with 10% fetal bovine serum, 1% penicillin–streptomycin, and 1% GlutaMax. Unless otherwise mentioned, v2L tau biosensor cells were used for all seeding assays. Details on these biosensors have been recently published [8]. Cell lines were checked for mycoplasma contamination (Venor-GEM Mycoplasma Detection kit).

### Proteomics screen

T225 flasks were coated with 10 mL of 0.01 mg/mL poly-D-lysine (PDL) for 3 h in the incubator and rinsed with PBS before plating cells. 22 million cells were plated in 25 ml/T225 flask and allowed to settle overnight. The following day, cells were treated with 50 nM tau + Lipofectamine-2000 complexes (or 50 nM α-synuclein + Lipofectamine as a negative control) which were incubated for 20 min at RT prior to addition to cells. Cells were incubated with the fibrils for 5 h. Thirty minutes before the 5 h time point, cells were treated with BP (biotin phenol) at a final concentration of 500 µM at 37ºC. At 5 h, cells were treated with H_2_O_2_ at a final concentration of 1 mM and the flasks were agitated at RT for 1 min. The biotinylation reaction was quenched with the quenching buffer followed by three additional rinses. Quenching buffer was also used to scrape the cells to collect the cell pellets. This buffer was prepared as previously described in the APEX2 labeling protocol [68].

### On-Bead trypsin digestion

Protein concentrations were normalized across all the samples (~ 1 mg of starting lysate) based on the Pierce 660 assay readings and protein abundances from shotgun proteomics analysis of trypsin digests of these samples by the UT Southwestern Proteomics Core Facility. Lysates (1 mg) were incubated with 250µL of magnetic streptavidin beads at 4ºC for overnight incubation ~ 16 h. The next day, the beads were concentrated by magnet, and washed 2 × with 200µL of 50 mM Tris–HCl pH 7.5 followed by 2 × with 2 M urea + 50 mM Tris–HCl pH 7.5. The beads were then incubated with 80µL 2 M urea + 100μL 0.5ug/µl trypsin + 20µL 10 mM DTT to achieve a final urea concentration of 1 mM and a ratio of 1: 20 for trypsin: lysate, for 1 h at 25ºC with shaking at 1000 rpm in a thermomixer. The beads were washed 2x with 60 µl of 2 M urea + 50 mM Tris–HCl pH 7.5 and the two washes were combined with the supernatant. The eluate was reduced with DTT at a net concentration of 4 mM by incubating for 30 min with shaking at 1000 rpm, 25ºC. The samples were alkylated with 10 mM iodoacetamide for 45 min at 25ºC with shaking at 1000 rpm.

50 mM Tris–HCl pH 7.5 was then added to achieve a final urea concentration of 0.73 M. Samples were incubated overnight (~ 15 h) at 37ºC with shaking at 1000 rpm to allow complete trypsin digestion. The samples were removed from the thermomixer and spun down. Trypsin was quenched by acidifying the samples to pH < 3 with the addition of formic acid at a final concentration of 1%.

### TMT Mass spectrometry

5μL of 10% trifluoroacetic acid (TFA) was added to each sample, and solid-phase extraction was performed on each sample using an Oasis HLB 96-well μElution plate (Waters). Eluates were dried and reconstituted in 50μL of 100 mM triethylammonium bicarbonate (TEAB). 10μL of each sample was labeled with 4μL of a different TMT10plex reagent (Thermo Scientific, label TMT10-131 not used). Samples were quenched with 1μL of hydroxylamine, mixed, and dried in a SpeedVac. Samples were reconstituted in 2% acetonitrile, 0.1% formic acid to a concentration of 0.5 ug/μL.

2μL of each TMT sample were injected onto an Orbitrap Fusion Lumos mass spectrometer coupled to an Ultimate 3000 RSLC-Nano liquid chromatography system. Samples were injected onto a 75 μm i.d., 75-cm long EasySpray column (Thermo) and eluted with a gradient from 0–28% buffer B over 180 min. Buffer A contained 2% (v/v) acetonitrile (ACN) and 0.1% formic acid in water, and buffer B contained 80% (v/v) ACN, 10% (v/v) trifluoroethanol, and 0.1% formic acid in water. The mass spectrometer was operated in positive ion mode with a source voltage of 1.8 kV and an ion transfer tube temperature of 275 °C. MS scans were acquired at 120,000 resolution in the Orbitrap and top speed mode was used for SPS-MS3 analysis with a cycle time of 2.5 s. MS2 was performed with CID with a collision energy of 35%. The top 10 fragments were selected for MS3 fragmentation using HCD, with a collision energy of 55%. Dynamic exclusion was set for 25 s after an ion was selected for fragmentation.

### Proteomics data analysis

Raw MS data files were analyzed using Proteome Discoverer v2.4 (Thermo), with peptide identification performed using Sequest HT searching against the human protein database from UniProt. Fragment and precursor tolerances of 10 ppm and 0.6 Da were specified, and three missed cleavages were allowed. Carbamidomethylation of Cys and TMT labeling of N-termini and Lys sidechains were set as a fixed modification, with oxidation of Met set as a variable modification. The false-discovery rate (FDR) cutoff was 1% for all peptides.

For every biological replicate, absolute abundance of each protein was first normalized to the total protein abundance of a particular lysate sample to account for any differences in total protein concentrations across samples before comparison. These values were used to calculate the relative enrichment of proteins specific to tau seeding: Protein Abundance Ratio = (sAPEX2 P301S + seeds)/(sAPEX2 P301S – seeds).

Normalized protein abundance ratios for sAPEX2 P301S and sAPEX2 alone (negative control) treated with and without tau fibrils were compared using unpaired t- test on three independent biological replicates; two-stage step-up (Benjamini, Krieger, and Yekutieli), FDR 1.00%). Positive relative abundance values on the graph indicate enrichment in the aggregation proteome. Statistical significance was determined based on q value. In the dataset, MAPT appears as a negatively enriched relative abundance value due to background signal from the sAPEX2 alone ratios (+ seeds /- seeds). This artifactual result was thus not displayed on the volcano plot.

### Generation of a WT tau biosensor cell line

FM5 CMV promoter plasmids containing the mCerulean3 and mRuby3 fluorophores were digested with Esp3I restriction enzyme. The 3R and 4R isoforms of WT tau aa 246–408 were cloned into the cut plasmids using Gibson assembly, respectively. Constructs were sequence verified using UTSW’s Sanger sequencing core and used for making lentivirus. HEK293T cells were transduced with lentivirus containing both constructs for 48 h after which single cells expressing both fluorophores were sorted into 96-well plates. Monoclonal lines were tested for lipofectamine-mediated seeding with AD brain homogenate and the most sensitive clone was selected.

### Generation of a TDP-43 biosensor cell line

FM5 CMV promoter plasmids containing the mClover3 and mRuby3 fluorophores were digested with Esp3I restriction enzyme. WT TDP-43 (aa 275–414 with the addition of an alanine after the start site and preceding the actual TDP43 sequence) was cloned into the cut plasmids using Gibson assembly to generate constructs expressing TDP-43 tagged to the respective fluorophores. Constructs were sequence verified using UTSW’s Sanger sequencing core and used for making lentivirus.

HEK293T cells were transduced with the TDP-43 lentivirus and were sorted into monoclonal cells for highest expression of the two fluorophores. Monoclonal cell line that responded with maximum seeding to different FTLD TDP-43 lysates was chosen for the seeding assay reported here. Cells expressing the individual fluorophores were also sorted as fluorophore compensation controls for detecting FRET on the flow cytometer.

### Biosensor seeding assay

All biosensor assays were performed with “naked seeding” (no cation-based transfection reagent). Cell lines were plated at a density of 15,000 cells/well of a 96 well plate and allowed to settle overnight. Cells were treated with an appropriate concentration of recombinant tau fibrils for 48 h, and then harvested for flow cytometry. Fibril preps were sonicated in a water bath sonicator for 1 min at 65Amp prior to cell treatment. Recombinant tau fibrils were prepared as previously described [28].

For seeding with brain lysates and for the α-synuclein biosensor seeding assay, 8,000 cells/well (10,000 cells/well for TDP-43 biosensors) were plated in 96 well plates and seeding was monitored for 72 h (or 5 days for WT tau biosensors). In the case of brain homogenates, biosensors were treated with 25 µg of tauopathy lysates and 10 µg of FTLD lysate. These were sonicated for 1 min at 65 Amp in a water bath sonicator. For seeding with α-synuclein, fibrils were sonicated for 5 min total, 1 min on /1 min off at 65 Amp, and used at a final concentration of 400 nM.

All seeding results are reported as % FRET + cells. The FRET data were plotted by subtracting the background signal (no exogenous tau added) which was negligible for all conditions (no FRET recorded in the absence of tau seeds), unless otherwise specified.

### Brain homogenization

Brain tissue from clinically and neuropathologically characterized cases of AD, CBD, and FTLD TDP-43 were obtained from UTSW and Washington University in St. Louis. All human tissues used in these experiments were derived from autopsy subjects. Since deceased subjects are not considered human subjects for research purposes, these studies were exempt from human subjects research regulations and did not require IRB approval. Brain samples were weighed and added to 1X TBS supplemented with cOmplete Ultra (Roche) protease inhibitor to prepare a 10% w/v solution. The brains were homogenized using a probe homogenizer to obtain a slurry that was sonicated for 15 min total, 1 min on/ 30 s off. The sonicated samples were centrifuged at 4C for 15 min at 21,300 × g. Protein concentration of the supernatant was measured using Pierce 660 assay and was subsequently used for naked seeding.

### Uptake assay

The uptake assay was performed as previously described [28]. v2L cells were plated overnight at a density of 8,000 cells/well of a 96 well plate. Cells were treated with 25 nM of AF-647 labeled tau fibrils or AF-647 dye alone as a negative control. After 4 h of incubation with the fibrils, cells were harvested with 0.25% trypsin for flow cytometry.

The labeled fibrils used in this assay were obtained by incubating recombinant tau fibrils (8 μM, 200μL) with lyophilized AF-647 dye (25 μg) for 1 h at room temperature (RT) followed by quenching the reaction with 100 mM glycine and subsequent dialysis in a 3500 kDa dialysis cassette.

The median fluorescence intensity (MFI) values representing the amount of tau internalized were plotted after subtracting the background MFI of the dye alone signal for all conditions. These MFI values were then normalized relative to the appropriate control condition of the respective experiment (DMSO, NTG, or Scr controls).

### Compound treatments

96 well plates were coated with 0.01 mg/mL PDL and incubated at 37C for 3 h followed by washout with PBS. v2L cells were plated at a density of 15,000 cells/well and allowed to settle overnight. Cells were treated with different compounds (ML-240, NMS-873, MG132, and LLOMe) for about an hour upon which 25 nM of recombinant tau fibrils were introduced to the media. After four hours of incubation with the fibrils (five hours with compounds), the media was replaced with fresh media and uptake or seeding was monitored for 4 h and 48 h, respectively.

### Flow cytometry

To harvest cells for flow cytometry, media was removed, and cells were treated with 0.05% trypsin (0.25% trypsin for uptake assay) for 5 min at 37C (0.25% trypsin, 15 min at 37C in case of PDL coated plates). Trypsin was quenched with cold media and cells were resuspended a few times before transferring the suspension to 96-well round-bottom plates which were centrifuged at 1000 rpm for 5 min. Supernatant was removed and the cell pellets were resuspended in 2% paraformaldehyde (PFA) and allowed to fix for 10 min at RT. Cells were spun down again, PFA was removed, and cells were resuspended in PBS and stored at 4C until ready to be run on a flow cytometer (BD LSRFortessa) for quantifying the FRET signal.

### Cloning

FM5 vector with UBC promoter was used to clone all the APEX constructs. sAPEX fragments (AP and EX) were PCR amplified from the constructs provided by the laboratory of Alice Ting (Stanford). Amplified sAPEX fragments were appended on to the c-terminus of RD tau fragments via a linker using overlap PCR. Using Gibson assembly, the final gene fragments were cloned into FM5 UBC plasmid which was cut with Esp3I. All Gibson reaction products were transformed into Stbl3 bacterial cells. Bacterial colonies were inoculated, DNA was purified using Qiagen miniprep kit, and the sequences were verified using Sanger sequencing at UTSW’s sequencing facility.

### Lentivirus production

Low passage HEK293T cells were plated at ~ 70% confluency in 6 well plates and allowed to settle overnight. A master mix was prepared using 400 ng of plasmid of interest, 400 ng of VSVG, and 1200 ng of PSP plasmids required for virus packaging, along with 7.5L of TransIT 293 and 120ul of OMEM per well of a 6 well plate. The master mix was allowed to incubate at RT for 30 min upon which it was added to the cells drop-wise. The virus was harvested 48 h later by collecting the media, centrifuging it for 5 min at 1000 rpm, and then freezing the aliquoted supernatant.

### Differentiation and culturing of human iPSC-derived cortical neurons

We utilized the integrated, inducible, and isogenic Ngn2 iPSC line (i^3^N). It was previously shown that expression of the transcription factor neurogenin-2 (Ngn2) induces rapid differentiation of iPSCs into cortical glutamatergic neurons [69]. This iPSC line harbors a doxycycline-inducible mouse Ngn2 transgene at an adeno-associated virus integration site 1 (AAVS1) safe-harbor locus, allowing for a simplified differentiation protocol [70, 71]. iPSCs were dissociated with Accutase (Sigma, A6964) and plated onto basement membrane extract-coated plates (R&D, 3434–001–02). Ngn2 expression was induced with 2 μM doxycycline hyclate (Sigma, D9891) in KSR media alone with 10 μM SB431442 (R&D, 1614), 2 μM XAV939 (Stemgent, 04–0046) and 100 nM LDN-193189 (Stemgent, 04–0074) (doxycycline hyclate is maintained in all medias going forward). On day 2, cells were fed with a 1:1 ratio of KSR media + SB/XAV/LDN and N2-supplmented neural induction media with 2 μg/ml puromycin (Life Technologies, A1113803). On day 3, cells were fed with N2-supplemented neural induction media. On day 4, cells were dissociated with Accutase and plated onto PDL-(Sigma, P1149) and laminin-(Life Technologies, 23 017–015) coated tissue culture plates. Cells were subsequently maintained with neurobasal media (Life Technologies, 21,103,049) supplemented with NeuroCult SM1 (StemCell Technologies, 05711) and 10 ng/ml brain-derived neurotrophic factor (R&D, 248-BD-005/CF) until collected.

### Compound treatment and seeding assay in neurons

Differentiated human neurons were treated with tau RD P301S lentivirus (tau-clover at an MOI of 3; tau-ruby at an MOI of 2). 48 h later, the transduced cells were simultaneously incubated with 15 nM FL, WT recombinant tau fibrils and the VCP inhibitors, ML-240 and NMS-873 at 100 nM for 48 h. For acute exposure with tau fibrils and the compounds, the neuronal biosensors were treated with 1 μM ML-240 and 100 nM NMS-873 for 4 h followed by media replacement. FRET signal, used as a measure of tau aggregation, was monitored over 48 h using ImageXpress Confocal HT.ai High-Content Imaging System (Molecular Devices). 4 images per well, with 5 wells per condition of a 96 well plate, were collected for the clover, ruby, and FRET channels. Single color controls were used to correct for fluorophore bleed through using an automated algorithm on MATLAB. Single color controls were used to measure the bleedthrough coefficient of each channel by fitting the correlation of pixel intensities in the single color channel and the “empty” channel with a linear fit. Images from the acceptor/donor channels of real data were then multiplied by the resulting slopes (i.e. bleedthrough coefficients) to approximate the amount of bleedthrough. Finally, FRET channel images were corrected by subtracting these bleedthrough images from the FRET images. The corrected images were analyzed to calculate the FRET area using an ImageJ macro and the quantified FRET area was plotted for different conditions.

### Imaging

All high magnification images were taken on the Nikon SoRA at 60x (oil immersion) magnification. For imaging, 96-well Cellvis glass bottom plates were coated with PDL followed by washout before plating cells of interest. Nuclei were stained with Hoechst (1 g/mL) for 10’ at 37C prior to fixing cells in 4% PFA for 10’ at RT. Cells were washed twice with PBS, 5’ each, and saved in PBS at 4C until imaged on the microscope.

### CRISPR/Cas9 Screen for VCP Cofactors

CRISPR constructs for the cofactors were outsourced to Twist Biosciences for synthesis. Constructs not synthesized by the company were cloned in the lab using standard ligation reactions. Four guides per gene were chosen from the Brunello library deposited online and ordered as duplex DNA from IDT. LentiCRISPRv2 (Addgene #52961) was cut using Esp3I, and T4 ligase was used for all ligation reactions of the guides into the plasmid. Stbl3 bacteria were transformed with the ligated products, selected colonies were inoculated, mini-prepped using Qiagen kit, and the purified DNA was sequence-verified. Pooled lentivirus was prepared with four constructs per gene and v2L cells were transduced with the virus at the desired MOI. After 24 h, cells were expanded in puromycin media (2μg/mL) for selection of the KO population. Selected populations were eventually used for seeding and uptake assays. The KO was verified using western blot.

### siRNA Knockdown

siRNAs were ordered from Origene. 300,000 v2L cells were plated in 6 well plates and allowed to settle overnight. The next day, cells were treated with 100 nM of each siRNA, with a total of three siRNAs per gene using RNAiMax Lipofectamine (Thermo) as a transfection vehicle at 7.5 µl/well. After 48 h of transfection, the cells were plated in 96 well plates for seeding and uptake assays and used for western blot to verify the knockdown.

### Western blot

Cell pellets were lysed in RIPA buffer and allowed to sit on ice for 5 min followed by a 15,000 g spin for 10 min at 4C. The supernatants were used to determine the protein concentrations using Pierce 660 assay. 15 μg of total protein was treated with SDS buffer + BME and heated at 95C for 10 min. Samples were loaded onto 4–12% bis–tris gels and the proteins were transferred onto nitrocellulose membranes using the Biorad turbo transfer machine. All incubations for subsequent steps were done in TBS + 0.05% Tween-20 (TBST). The membranes were first incubated in blocking buffer (5% milk powder + TBST) for 1 h at RT, followed by primary antibodies in the blocking buffer at 4C with overnight shaking. After the primary antibody incubation, the membranes were washed 3x with TBST, 10 min each. Then, appropriate HRP-conjugated secondary antibodies in blocking buffer were added to the membranes for a 1.5 h incubation at RT. Membranes were again washed 3 × in TBST followed by a single wash in TBS alone before reading the HRP signal using the Thermo ECL kit.

### Statistical analysis

Proteomics data was analyzed using unpaired t- test, two-stage step-up (Benjamini, Krieger, and Yekutieli), FDR 1.00%. One-Way ANOVA (Šídák method) with a 95% confidence interval was used for all the other statistical analyses unless otherwise stated. Paired t-test was used to analyze the neuron data.

For all seeding experiments, one representative experiment of three biological replicates is presented. Each experiment includes technical triplicates. Thus, the seeding data shows S.D. Uptake assay data was normalized across all three biological replicates and is therefore presented with S.E.M.

The P values are described as follows: ns = not significant/ *P* > 0.05, * = *P* ≤ 0.05, ** = *P* ≤ 0.01, *** = *P* ≤ 0.001, **** = *P* ≤ 0.0001.

### Graphics

Biorender.com was used to create the graphics presented here and has granted publication and licensing rights (agreement number: RX26PLQVWQ).

**Table:** **List of Reagents**

**Reagent**	**Vendor**	**Catalog No**
Acetonitrile ≥ 99.9%, LC–MS Reagent for LC–MS, for HPLC	Avantor	9829–03
Ammonium bicarbonate,ReagentPlus®, ≥ 99.0%	Sigma-Aldrich	A6141-500G
Anti-FAF2 Rabbit Polyclonal Antibody, Size = 150 µL	Fisher Scientific	16,251–1-AP
Anti-OTUB1 antibody [EPR13028(B)] (ab175200)	Abcam	ab175200
Anti-UBE4B antibody [EPR7471] (ab126759)	Abcam	ab126759
Anti-UBXD7 Antibody	EMD Millipore	AB10037
Anti-VCP antibody (ab11433)	Abcam	ab11433
Ataxin 3 Antibody	Fisher Scientific	702,788
ATXN3 (Human)—3 unique 27mer siRNA duplexes—2 nmol each	OriGene	SR302905
Benzonase Nuclease, ≥ 250 units/muL, ≥ 90% (SDS-PAGE)	Sigma-Aldrich	E1014-5KU
Biotinyl Tyramide, Tocris, 6241	R&D Systems	6241/25
Biotinyl tyramide, ≥ 97% (HPLC)	Sigma-Aldrich	SML2135-50MG
BME, ≥ 99.0%	Sigma-Aldrich	M6250-100ML
96 well glass bottom plates	Cellvis	P96-1.5H-N
Complete™, Mini, EDTA-free Protease Inhibitor Cocktail	Sigma-Aldrich	4,693,159,001
Corning 225cm^2^ Angled Neck Cell Culture Flask with Vent Cap	Corning	431,082
FAF2 (Human)—3 unique 27mer siRNA duplexes—2 nmol each	OriGene	SR308083
Formic acid 50ML UN 1779 3(8) / PGII	Sigma-Aldrich	56,302-50ML-GL
GAPDH Antibody (1D4)	Fisher Scientific	NB300-221
Gibson Assembly Master Mix	New England Biolabs	E2611S
Hoechst 33,342	Thermo Scientific	H3569
Invitrogen novex NuPAGE 4 12% Bis Tris Protein Gels, 1.0 mm, 10 well	Thermo Scientific	NP0321BOX
Iodoacetamide,single use vial of 56 mg	Sigma-Aldrich	A3221-10VL
Iproof™ High-Fidelity PCR Kit, 200 U (2 U/µl), 100 µl 1,725,331	Bio-Rad	1,725,331
Jumpstart™ Taq DNA Polymerase,with MgCl2	Sigma-Aldrich	D9307-50UN
Laemmli Sample Buffer 2X	Bio-Rad	1,610,737
Laemmli Sample Buffer, 4X	Bio-Rad	1,610,747
Lipofectamine RNAiMAX Transfection Reagent	Fisher Scientific	13–778-075
MG-132 25 mg	Fisher Scientific	S2619
Ml240, ≥ 98% (hplc)	Sigma-Aldrich	SML1071-5MG
NEBuilder HiFi DNA Assembly Master Mix—10 reactions	New England Biolabs	E2621S
NGLY1 (Human)—3 unique 27mer siRNA duplexes—2 nmol each	OriGene Technologies	SR310927
NGLY1 Polyclonal Antibody	Thermo Scientific	A305-547A-T
NheI-HF® Restriction Enzyme	New England Biolabs	R3131S
NMS-873	MedChem Express	HY-15713
Npl4 Antibody	Cell Signaling Technology	13489S
NPLOC4 (Human)—3 unique 27mer siRNA duplexes—2 nmol each	OriGene	SR310841
NSFL1C (Human)—3 unique 27mer siRNA duplexes—2 nmol each	OriGene	SR311050
Nsfl1c Polyclonal Antibody	Fisher Scientific	PA5-21,633
Nupage™ 4 12% Bis Tris Protein Gels, 1.5 mm, 15 well	Fisher Scientific	NP0336BOX
Nupage™ 4–12% Bis–Tris Protein Gels, 1.5 mm, 10-well	Fisher Scientific	NP0335BOX
One Shot Stbl3 Chemically Competent	Thermo Scientific	C737303
Opti-MEM™ I Reduced Serum Medium	Fisher Scientific	31–985-070
Opti-MEM™ I Reduced Serum Medium	Thermo Fisher Scientific	31,985,070
Pierce™ 660 nm Protein Assay	Fisher Scientific	22,660
Pierce™ BCA® Bovine Serum Albumin Standard Set	Thermo Scientific	23,208
Pierce™ Nitrocellulose Membranes, Thermo Scientific, Roll	Fisher Scientific	88–018
Pierce™ Streptavidin Magnetic Beads	Thermo Fisher Scientific	88,817
PLAA (Human)—3 unique 27mer siRNA duplexes—2 nmol each	OriGene	SR306209
Poly-D-lysine hydrobromide,mol wt 70,000–150,000, lyophilized powder	Sigma-Aldrich	P6407-5MG
Precision Plus Protein™ Dual Color Standards, 10–250 kDa	Bio-Rad	1,610,374
QIAprep Spin Miniprep Kit (250)	Qiagen	27,106
Redtaq® ReadyMix™ PCR Reaction Mix,Complete PCR reagent	Sigma-Aldrich	R2523-100RXN
RPS27A Human siRNA Oligo Duplex (Locus ID 6233)	OriGene	SR304187
S.O.C. Medium	Thermo Fisher Scientific	15,544,034
Sequencing Grade Modified Trypsin, Promega	Promega	V5111
Sodium Ascorbate, Powder, USP, Packaging = Poly Bottle, Size = 100 g	Spectrum Chemical	S1349-100GM
SVIP (Human)—3 unique 27mer siRNA duplexes—2 nmol each	OriGene	SR316907
SYVN1 Human siRNA Oligo Duplex (Locus ID 84447)	OriGene	SR325336
T4 DNA Ligase	New England Biolabs	M0202L
Thermo Scientific Pierce DTT (Dithiothreitol)	Thermo Fisher Scientific	20,290
Thermo Scientific 6X DNA Loading Dye	Thermo Fisher Scientific	R0611
Thermo Scientific FastDigest BsmBI (Esp3I) Promotion	Thermo Fisher Scientific	FD0454
Thermo Scientific Pierce 660 nm Protein Assay	Fisher Scientific	PI22660
Trans-Blot, 1,704,270	Bio-Rad	1,704,270
Trans-Blot® Turbo™ RTA Midi Nitrocellulose Transfer Kit, for 40 blots	Bio-Rad	1,704,271
TransIT®−293 Transfection Reagent	Fisher Scientific	MIR 2700
Trolox	Sigma-Aldrich	238,813-5G
Tween® 20, viscous liquid, CAS 9005–64-5, Sigma-Aldrich P1379-1L	Sigma-Aldrich	P1379-1L
UBE4B (Human)—3 unique 27mer siRNA duplexes—2 nmol each	OriGene	SR306958
UBXN6 (Human)—3 unique 27mer siRNA duplexes—2 nmol each	OriGene	SR312922
UBXN6 Polyclonal Antibody	Thermo Scientific	PA5-84,520
Ufd1 Antibody	Cell Signaling Technology	13789S
UFD1L (Human)—3 unique 27mer siRNA duplexes—2 nmol each	OriGene	SR305021
VCP (Human)—3 unique 27mer siRNA duplexes—2 nmol each	OriGene	SR322176
Venor™ GeM Mycoplasma Detection Kit, PCR-based	Sigma-Aldrich	MP0025-1KT
Vinculin Antibody	Fisher Scientific	NBP2-41,237

## Supplementary Information

Supplementary Material 1: Supplemental Figure 1. VCP identified by proximity labeling from tau aggregation. **(A)** Western blot probed for biotin using streptavidin-HRP showed the earliest reconstitution of P301S tau-sAPEX2 activity at 5h.

Supplementary Material 2: Supplemental Figure 2. Reduction of VCP inhibits tau seeding. **(A)** Western blot showing KD of VCP compared to scrambled (Scr) control siRNA-treated cells. **(B)** Images representing tau-clover signal. Scale bar = 50μm. VCP KD cells are brighter but fewer in number due to reduced cell proliferation. **(C) **Flow plots depicting a shift in dual positive biosensor population in quadrant 2 (Q2) for the VCP KD cell line, highlighting the increase in fluorescence levels of the biosensors as observed under the microscope. The population size, however, is reduced, indicating reduced cell proliferation. **(D) **VCP KD cells are brighter as depicted by the higher GFP MFI values. Graph is representative of n=3 independent experiments, with each data point derived from technical triplicate. Error bars represent S.D. One-Way ANOVA with a 95% confidence interval. P value: **** < 0.0001. **(E) **Flow plots showing no background spontaneous seeding (FRET+ values) in the VCP KD cells in the absence of exogenous tau fibrils, despite the increased basal fluorescence. **(F) **Aggregates in the VCP KD cells are larger and brighter as depicted by the higher FRET MFI values. Graph is representative of n=3 independent experiments, with each data point derived from technical triplicate. Error bars represent S.D. One-Way ANOVA with a 95% confidence interval. P value: **** < 0.0001.

Supplementary Material 3: Supplemental Figure 3. ML-240 increases tau aggregation and its kinetics.** (A) **Higher magnification (60x, oil immersion) images show no aggregates or puncta in the absence of tau fibrils under all conditions. ML-240 and MG132 increase, whereas NMS-873 decreases tau aggregation. Scale bar = 25μm. **(B) **ML-240 increased the kinetics of tau seeding with FRET signal detectable by 8 h.

Supplementary Material 4: Supplemental Figure 4. ML-240 induces Gal3 puncta formation. U2OS cells overexpressing mRuby3-galectin3 were treated with different compounds for 5h. **(A) **ML-240 (3μM) and LLOMe (1mM) induced Gal3 puncta, while NMS-873 (3μM) did not. Co-treatment of ML-240 and NMS-873 also induced Gal3 puncta. Representative images of n=3 independent experiments. Scale bar = 25μm.

Supplementary Material 5: Supplemental Figure 5. VCP inhibition impacts tau seeding early in the process. **(A) **ML-240 increased, and NMS-873 decreased tau aggregation only when administered <8h of seed exposure as represented by the tau-clover images at the indicated time points. Scale bar = 50μm.

Supplementary Material 6: Supplemental Figure 6. VCP inhibitors differentially impact tau seeding in human neurons. **(A)** Differentiated iPSC human neurons were transduced with tau RD (P301S)-clover/ruby lentivirus for 48h followed by seeding in the presence or absence of VCP inhibitors. Neurons were co-treated with tau fibrils and inhibitors for 4h prior to media replacement. FRET signal was measured at 48h. **(B)** Acute exposure with ML-240 (1μM) enhanced seeded tau aggregation whereas **(C)** NMS-873 (100nM) reduced tau aggregation. Error bars represent S.D. Representative data of n=3 independent experiments. Each dot represents an image taken per condition, with 4 different locations captured per well, for a total of 5 wells per condition. P values: **** < 0.0001, ** 0.0017; Paired t-test with a 95% confidence interval. **(D) **Representative images show the FRET signal indicative of seeding under different conditions in neurons. Scale bar = 100μm.

Supplementary Material 7: Supplemental Figure 7. ML-240 enhances seeding by recombinant α-synuclein. α-syn (A53T)-CFP/YFP biosensors were seeded with recombinant synuclein fibrils. **(A) **ML-240 increased α-synuclein seeding. No seeding was observed in the absence of compound. Representative data for n=3 independent experiments, with each data point derived from technical triplicate. Error bars represent S.D. One-Way ANOVA with a 95% confidence interval. P value, **** <0.0001. **(B) **Representative fluorescence microscopy images for effects of ML-240 on α-synuclein seeding. Scale bar = 50μm.

Supplementary Material 8: Supplemental Figure 8. VCP cofactors differentially regulate tau seeding.** (A) **Graphs representing the % FRET+ signal for cofactors (KO and KD) that did not affect tau seeding. **(B)** KO of UBXN6 increased tau seeding but the effect was most pronounced at higher tau concentrations. Graph represents n=3 independent experiments, with each data point derived from technical triplicate. Error bars represent S.D. One-Way ANOVA with a 95% confidence interval. P value, ****<0.0001. **(C)** KO of FAF2 caused spontaneous aggregation as observed by tau-clover puncta. Scale bar = 50μm. **(D) **Flow plots depict spontaneous aggregation in the FAF2 KO cells in the absence of exogenously added tau seeds as recorded by the FRET signal. **(E) **Western blots indicate absence of FAF2, ATXN3, NSFL1C, and UBE4B in respective knockout cells lines. Non-targeting guide (NTG) was used as a negative control. **(F) **Representative images showing increased basal fluorescence in NPLOC4 KD biosensors. Scale bar = 50μm. **(G)** Flow plots depict a shift in the dual positive biosensor population in quadrant 2 (Q2) for the NPLOC4 KD cell line indicating the increase in fluorescence levels of the biosensors as also observed under the microscope. **(H) **Flow plots indicate no background spontaneous aggregation in the NPLOC4 KD cells in the absence of exogenous tau fibrils, despite the increase in basal fluorescence. **(I)** Western blots indicate reduced protein levels of the cofactors in their respective KD cell lines. Scrambled siRNA (Scr) treated cell line was a negative control. **(J) **Representative images showing absence of any basal mRuby3-Gal3 puncta, either in the presence or absence of tau fibrils, in the FAF2 and UBXN6 cofactor KO cell lines. Scale bar = 25μm.

Supplementary Material 9: Supplemental Table 1.

## Data Availability

Data generated in this study but not presented here are available from the corresponding author on request.

## Abbreviations

VCP/p97: Valosin containing protein
sAPEX2: Split ascorbate peroxidase 2
RD: Repeat domain
FL: Full length
WT: Wild type
BP: Biotin-phenol
TMT-MS: Tandem mass-tag mass spectrometry
KO: Knockout
KD: Knockdown
AF-647: Alexa fluor-647
AD: Alzheimer’s disease
CBD: Corticobasal degeneration
TDP-43: TAR DNA-binding protein 43
