# Supplementary figures and images for: VCP regulates early tau seed amplification via specific cofactors
Source: Mol Neurodegener. 2025 Jan 7;20:2. doi: 10.1186/s13024-024-00783-z (PMC11707990; doi:10.1186/s13024-024-00783-z)

**A**

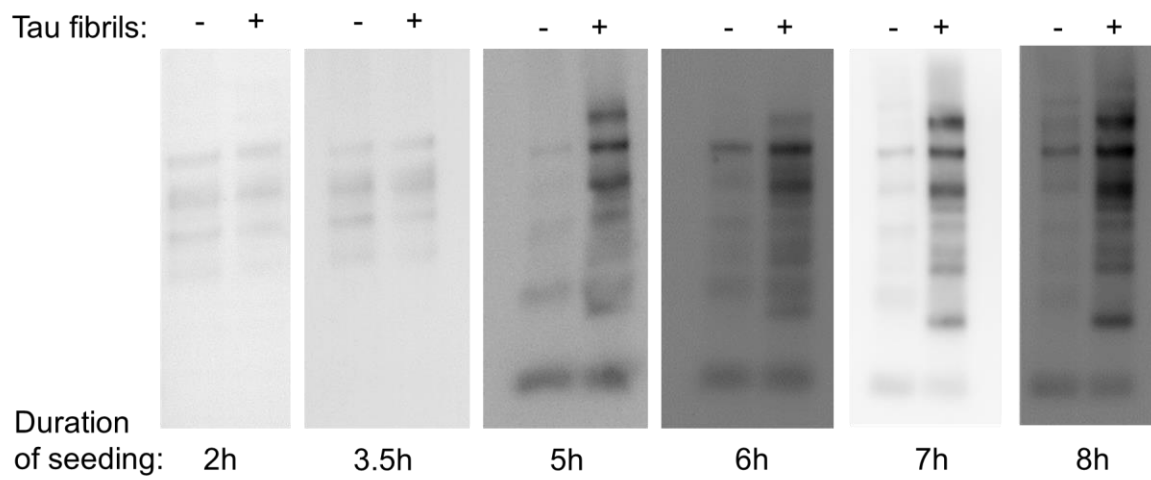

Supplement: Supplementary file 1 — Supplementary Material 1: Supplemental Figure 1. VCP identified by proximity labeling from tau aggregation. (A) Western blot probed for biotin using streptavidin-HRP showed the earliest reconstitution of P301S tau-sAPEX2 activity at 5h. [file 13024_2024_783_MOESM1_ESM.pdf]

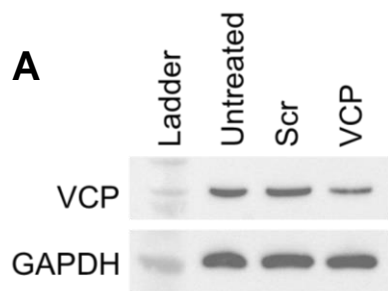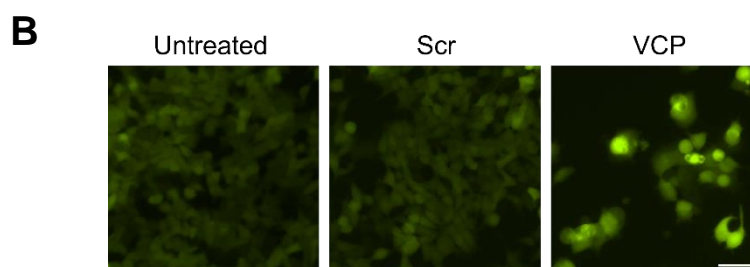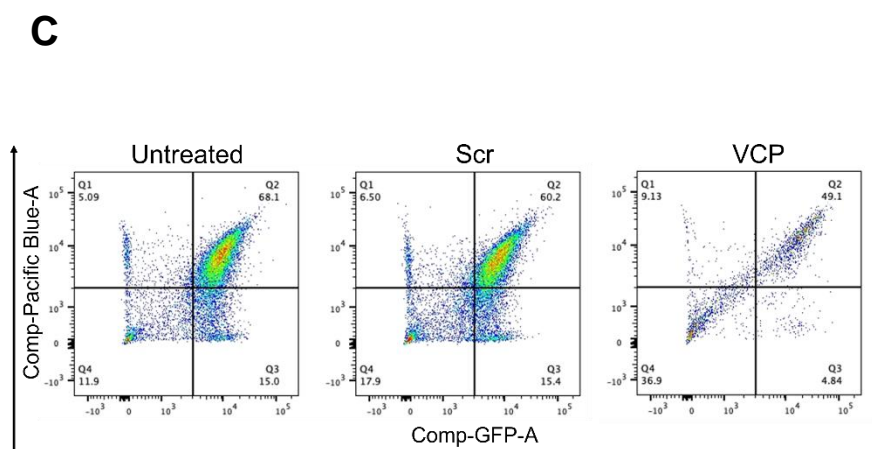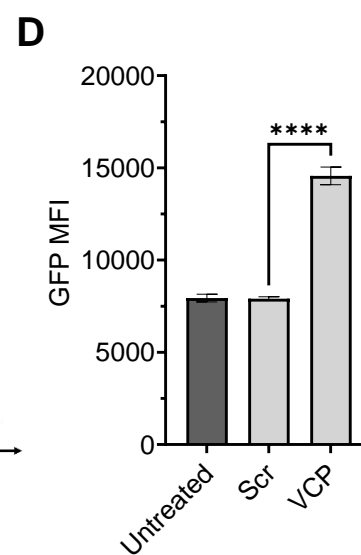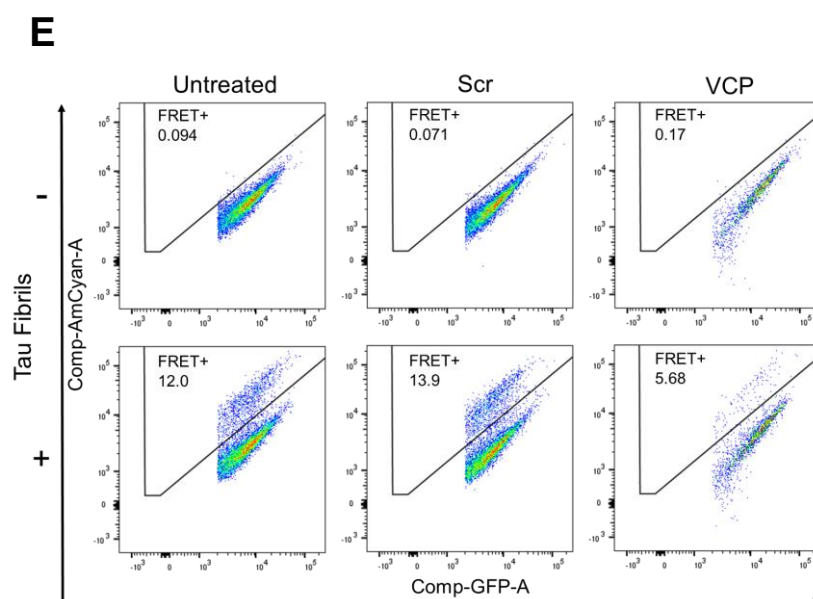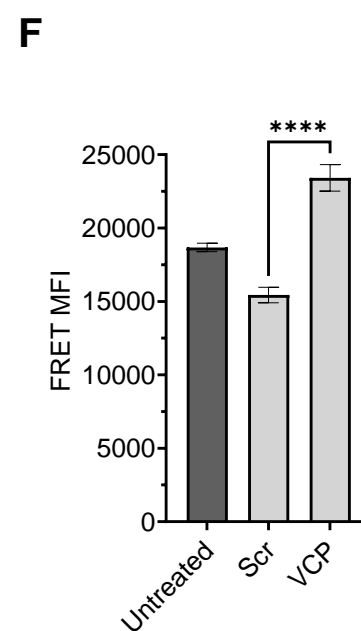

Supplement: Supplementary file 2 — Supplementary Material 2: Supplemental Figure 2. Reduction of VCP inhibits tau seeding. (A) Western blot showing KD of VCP compared to scrambled (Scr) control siRNA-treated cells. (B) Images representing tau-clover signal. Scale bar = 50μm. VCP KD cells are brighter but fewer in number due to reduced cell proliferation. (C) Flow plots depicting a shift in dual positive biosensor population in quadrant 2 (Q2) for the VCP KD cell line, highlighting the increase in fluorescence levels of the biosensors as observed under the microscope. The population size, however, is reduced, indicating reduced cell proliferation. (D) VCP KD cells are brighter as depicted by the higher GFP MFI values. Graph is representative of n=3 independent experiments, with each data point derived from technical triplicate. Error bars represent S.D. One-Way ANOVA with a 95% confidence interval. P value: **** < 0.0001. (E) Flow plots showing no background spontaneous seeding (FRET+ values) in the VCP KD cells in the absence of exogenous tau fibrils, despite the increased basal fluorescence. (F) Aggregates in the VCP KD cells are larger and brighter as depicted by the higher FRET MFI values. Graph is representative of n=3 independent experiments, with each data point derived from technical triplicate. Error bars represent S.D. One-Way ANOVA with a 95% confidence interval. P value: **** < 0.0001. [file 13024_2024_783_MOESM2_ESM.pdf]

**A**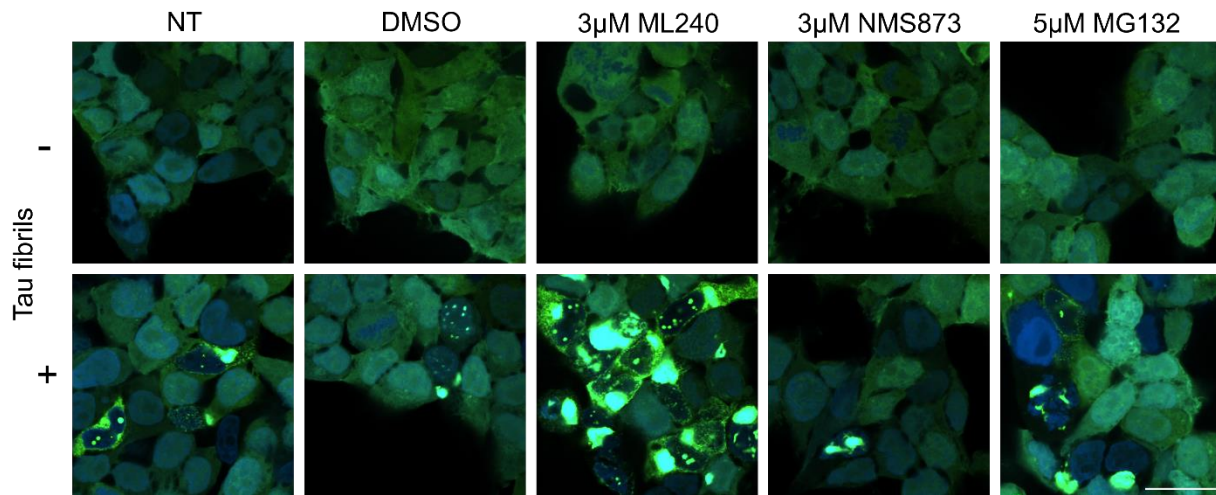**B**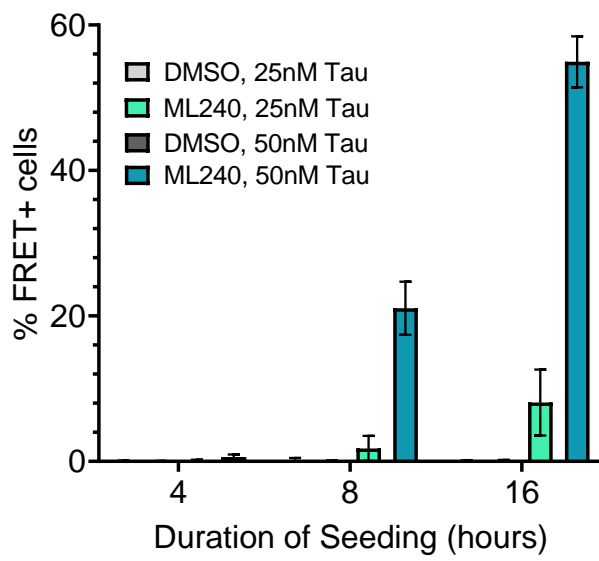

Supplement: Supplementary file 3 — Supplementary Material 3: Supplemental Figure 3. ML-240 increases tau aggregation and its kinetics. (A) Higher magnification (60x, oil immersion) images show no aggregates or puncta in the absence of tau fibrils under all conditions. ML-240 and MG132 increase, whereas NMS-873 decreases tau aggregation. Scale bar = 25μm. (B) ML-240 increased the kinetics of tau seeding with FRET signal detectable by 8 h. [file 13024_2024_783_MOESM3_ESM.pdf]

A

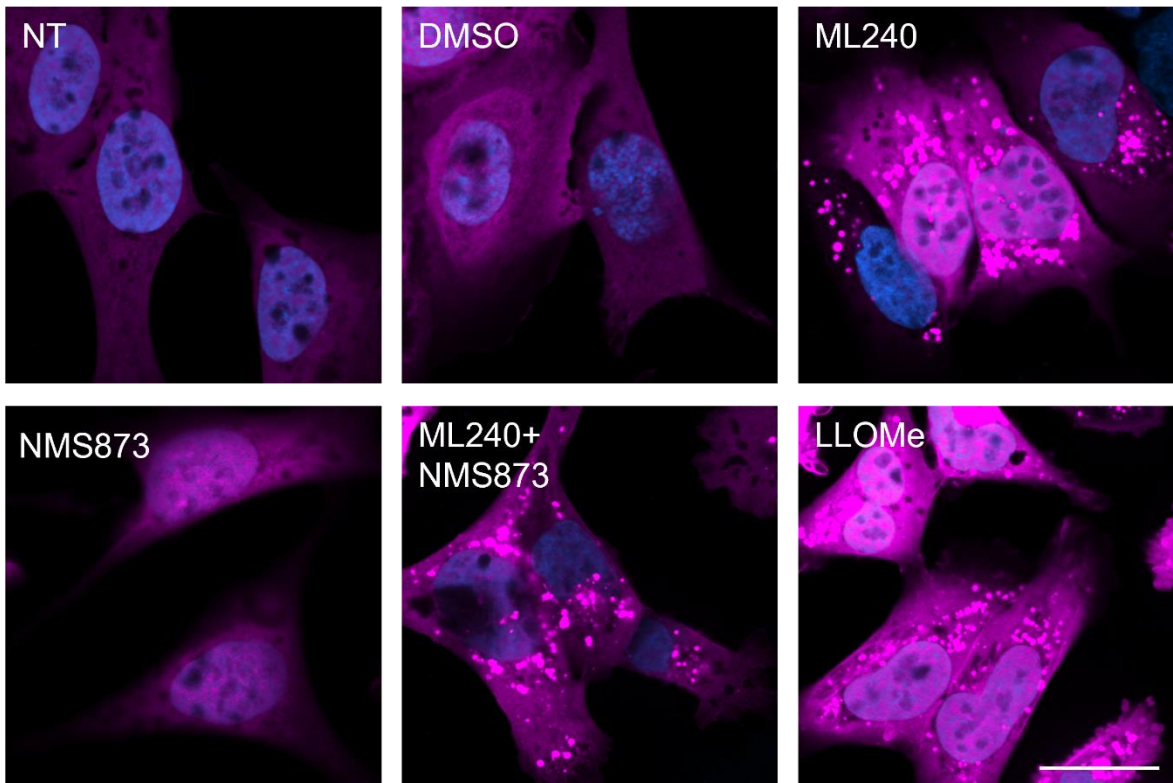

Supplement: Supplementary file 4 — Supplementary Material 4: Supplemental Figure 4. ML-240 induces Gal3 puncta formation. U2OS cells overexpressing mRuby3-galectin3 were treated with different compounds for 5h. (A) ML-240 (3μM) and LLOMe (1mM) induced Gal3 puncta, while NMS-873 (3μM) did not. Co-treatment of ML-240 and NMS-873 also induced Gal3 puncta. Representative images of n=3 independent experiments. Scale bar = 25μm. [file 13024_2024_783_MOESM4_ESM.pdf]

**A**

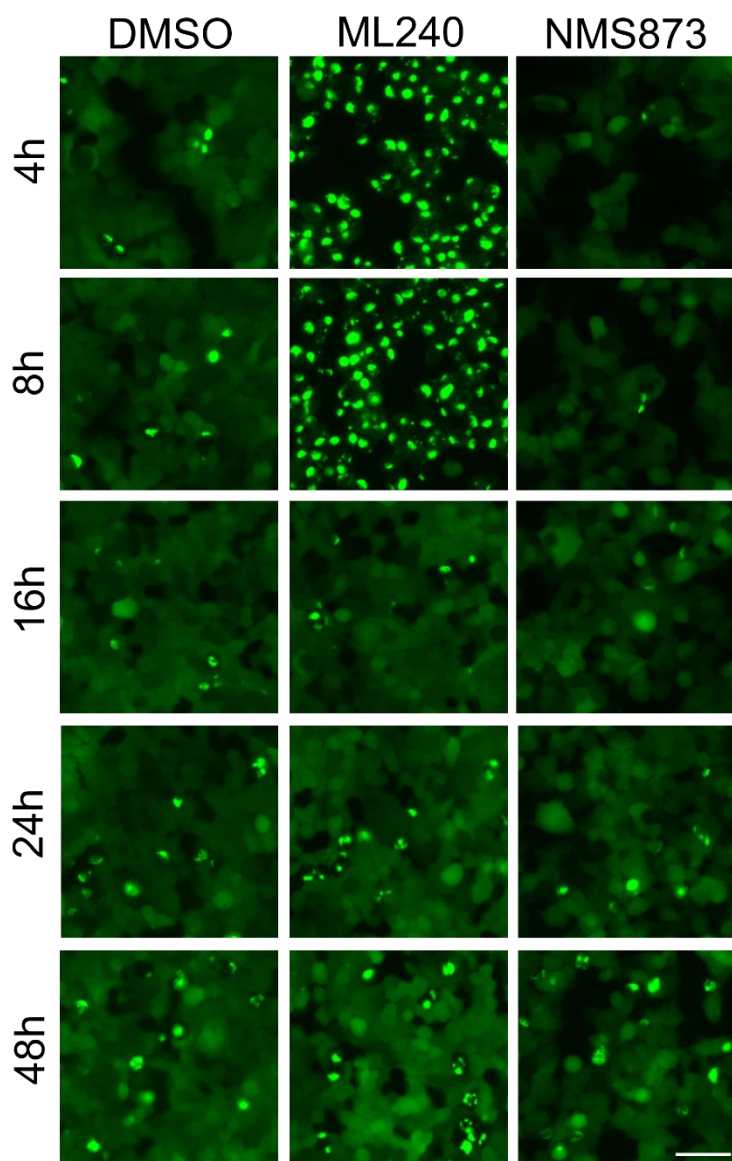

Supplement: Supplementary file 5 — Supplementary Material 5: Supplemental Figure 5. VCP inhibition impacts tau seeding early in the process. (A) ML-240 increased, and NMS-873 decreased tau aggregation only when administered <8h of seed exposure as represented by the tau-clover images at the indicated time points. Scale bar = 50μm. [file 13024_2024_783_MOESM5_ESM.pdf]

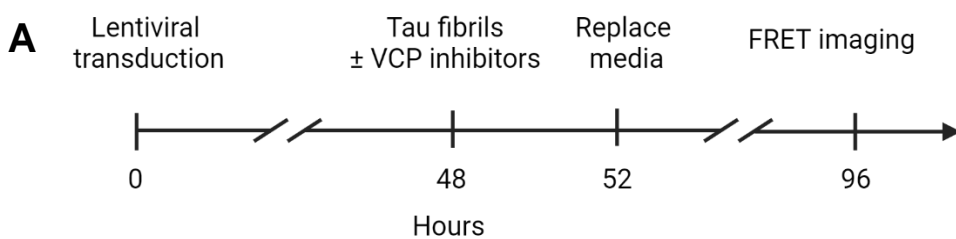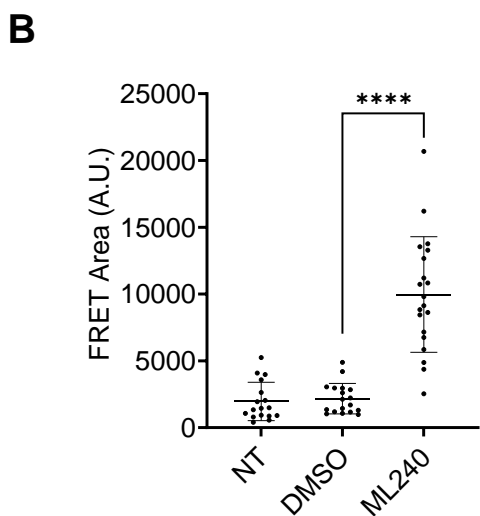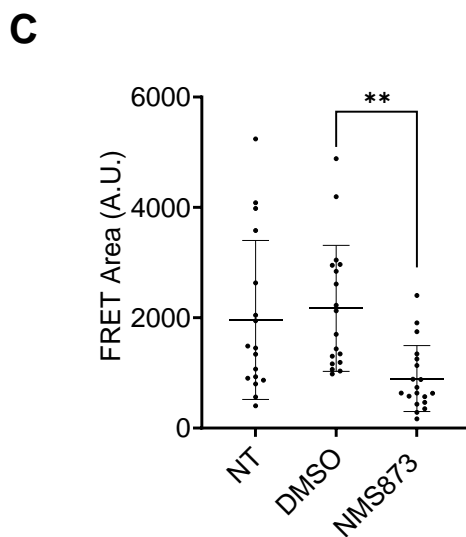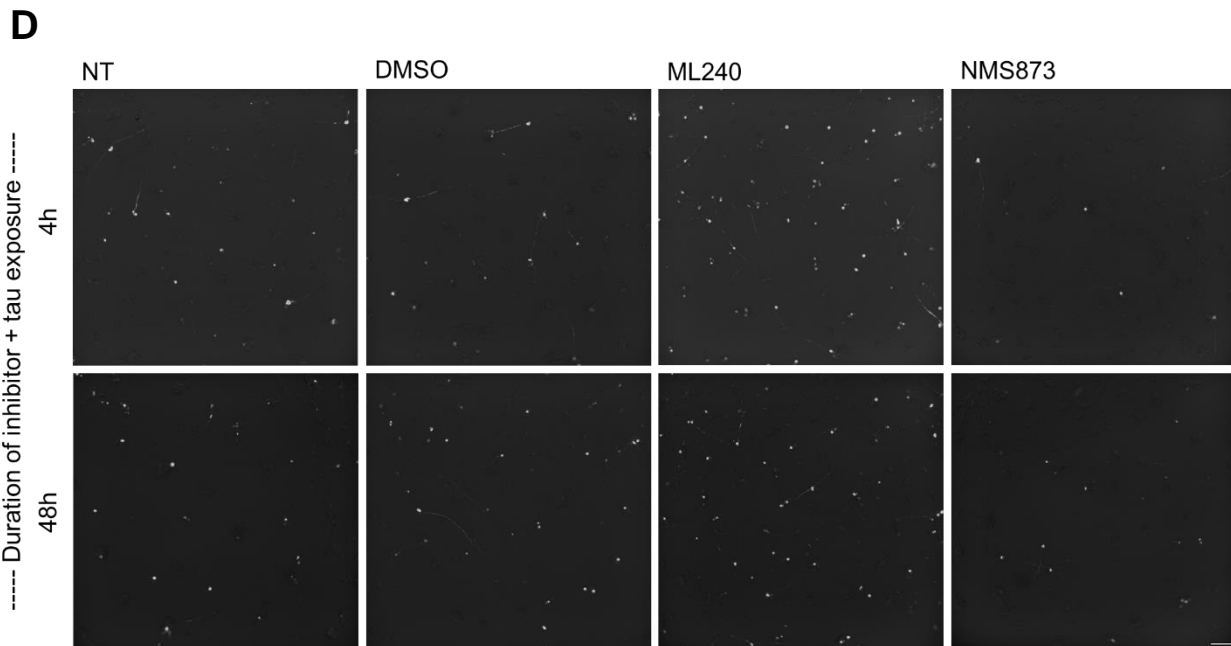

Supplement: Supplementary file 6 — Supplementary Material 6: Supplemental Figure 6. VCP inhibitors differentially impact tau seeding in human neurons. (A) Differentiated iPSC human neurons were transduced with tau RD (P301S)-clover/ruby lentivirus for 48h followed by seeding in the presence or absence of VCP inhibitors. Neurons were co-treated with tau fibrils and inhibitors for 4h prior to media replacement. FRET signal was measured at 48h. (B) Acute exposure with ML-240 (1μM) enhanced seeded tau aggregation whereas (C) NMS-873 (100nM) reduced tau aggregation. Error bars represent S.D. Representative data of n=3 independent experiments. Each dot represents an image taken per condition, with 4 different locations captured per well, for a total of 5 wells per condition. P values: **** < 0.0001, ** 0.0017; Paired t-test with a 95% confidence interval. (D) Representative images show the FRET signal indicative of seeding under different conditions in neurons. Scale bar = 100μm. [file 13024_2024_783_MOESM6_ESM.pdf]

**A**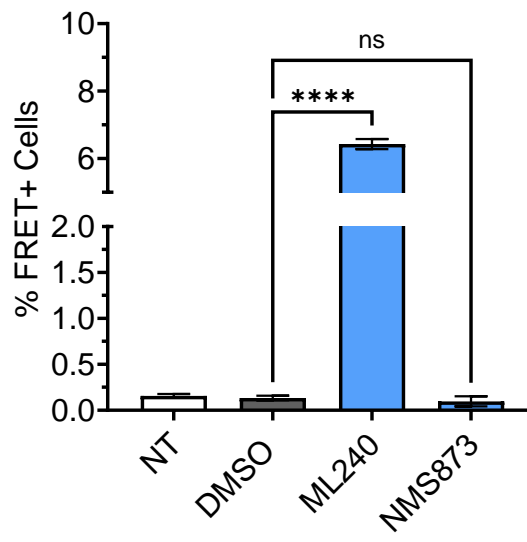**B**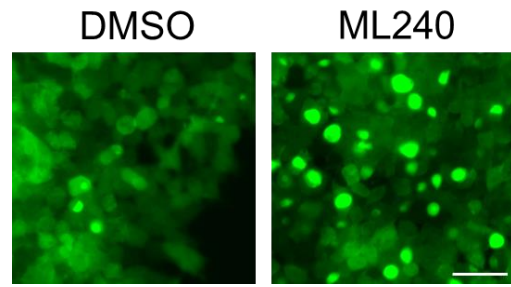

Supplement: Supplementary file 7 — Supplementary Material 7: Supplemental Figure 7. ML-240 enhances seeding by recombinant α-synuclein. α-syn (A53T)-CFP/YFP biosensors were seeded with recombinant synuclein fibrils. (A) ML-240 increased α-synuclein seeding. No seeding was observed in the absence of compound. Representative data for n=3 independent experiments, with each data point derived from technical triplicate. Error bars represent S.D. One-Way ANOVA with a 95% confidence interval. P value, **** <0.0001. (B) Representative fluorescence microscopy images for effects of ML-240 on α-synuclein seeding. Scale bar = 50μm. [file 13024_2024_783_MOESM7_ESM.pdf]
