# Supplementary material for: VCP regulates early tau seed amplification via specific cofactors
Source: Mol Neurodegener. 2025 Jan 7;20:2. doi: 10.1186/s13024-024-00783-z (PMC11707990; doi:10.1186/s13024-024-00783-z)
Supplement: Supplementary file 8 — Supplementary Material 8: Supplemental Figure 8. VCP cofactors differentially regulate tau seeding. (A) Graphs representing the % FRET+ signal for cofactors (KO and KD) that did not affect tau seeding. (B) KO of UBXN6 increased tau seeding but the effect was most pronounced at higher tau concentrations. Graph represents n=3 independent experiments, with each data point derived from technical triplicate. Error bars represent S.D. One-Way ANOVA with a 95% confidence interval. P value, ****<0.0001. (C) KO of FAF2 caused spontaneous aggregation as observed by tau-clover puncta. Scale bar = 50μm. (D) Flow plots depict spontaneous aggregation in the FAF2 KO cells in the absence of exogenously added tau seeds as recorded by the FRET signal. (E) Western blots indicate absence of FAF2, ATXN3, NSFL1C, and UBE4B in respective knockout cells lines. Non-targeting guide (NTG) was used as a negative control. (F) Representative images showing increased basal fluorescence in NPLOC4 KD biosensors. Scale bar = 50μm. (G) Flow plots depict a shift in the dual positive biosensor population in quadrant 2 (Q2) for the NPLOC4 KD cell line indicating the increase in fluorescence levels of the biosensors as also observed under the microscope. (H) Flow plots indicate no background spontaneous aggregation in the NPLOC4 KD cells in the absence of exogenous tau fibrils, despite the increase in basal fluorescence. (I) Western blots indicate reduced protein levels of the cofactors in their respective KD cell lines. Scrambled siRNA (Scr) treated cell line was a negative control. (J) Representative images showing absence of any basal mRuby3-Gal3 puncta, either in the presence or absence of tau fibrils, in the FAF2 and UBXN6 cofactor KO cell lines. Scale bar = 25μm. [file 13024_2024_783_MOESM8_ESM.pdf]

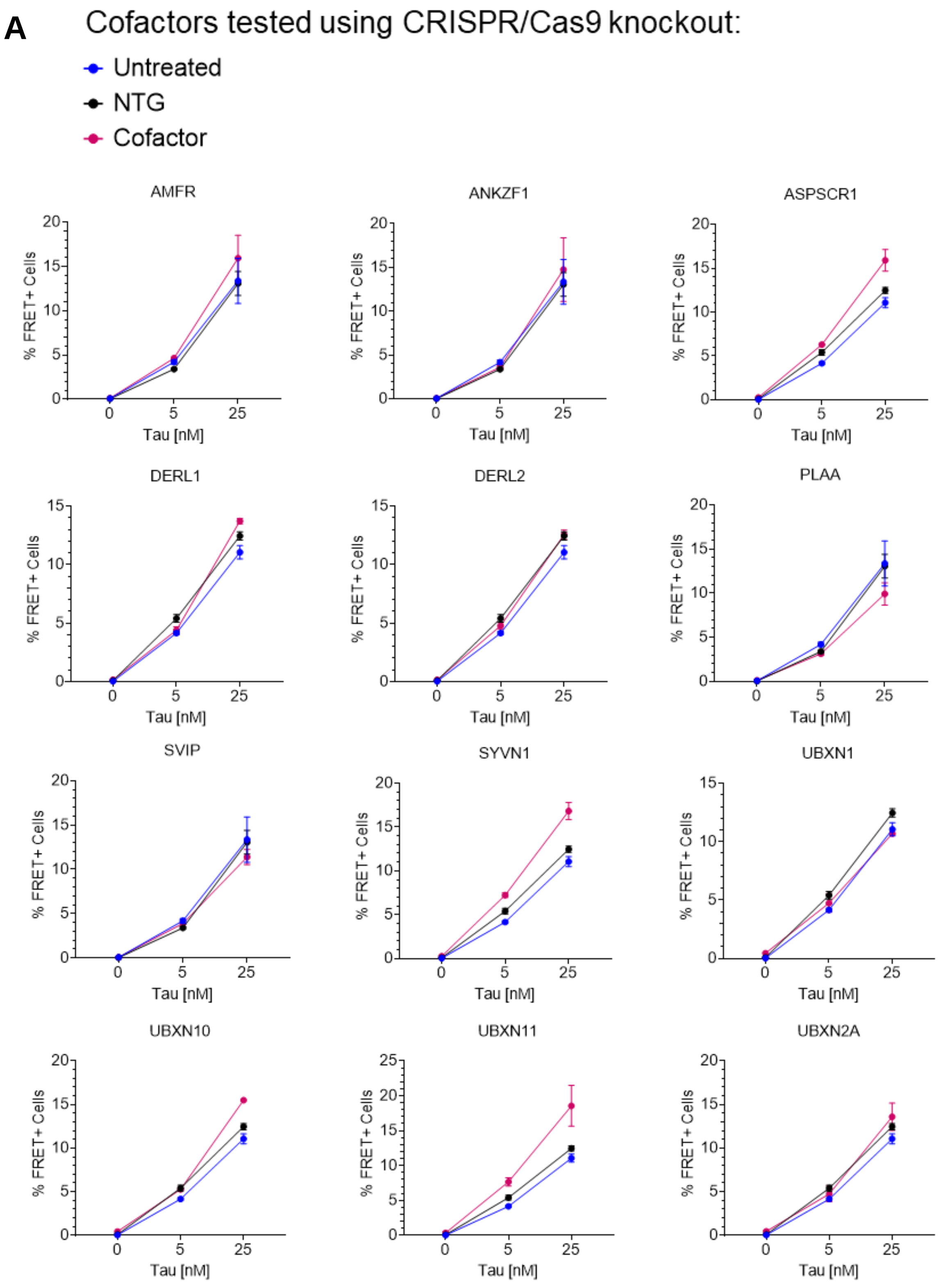
A
Cofactors tested using CRISPR/Cas9 knockout:
Untreated
NTG
Cofactor
AMFR
ANKZF1
ASPSCR1
DERL1
DERL2
PLAA
SVIP
SYVN1
UBXN1
UBXN10
UBXN11
UBXN2A
% FRET+ Cells
Tau [nM]

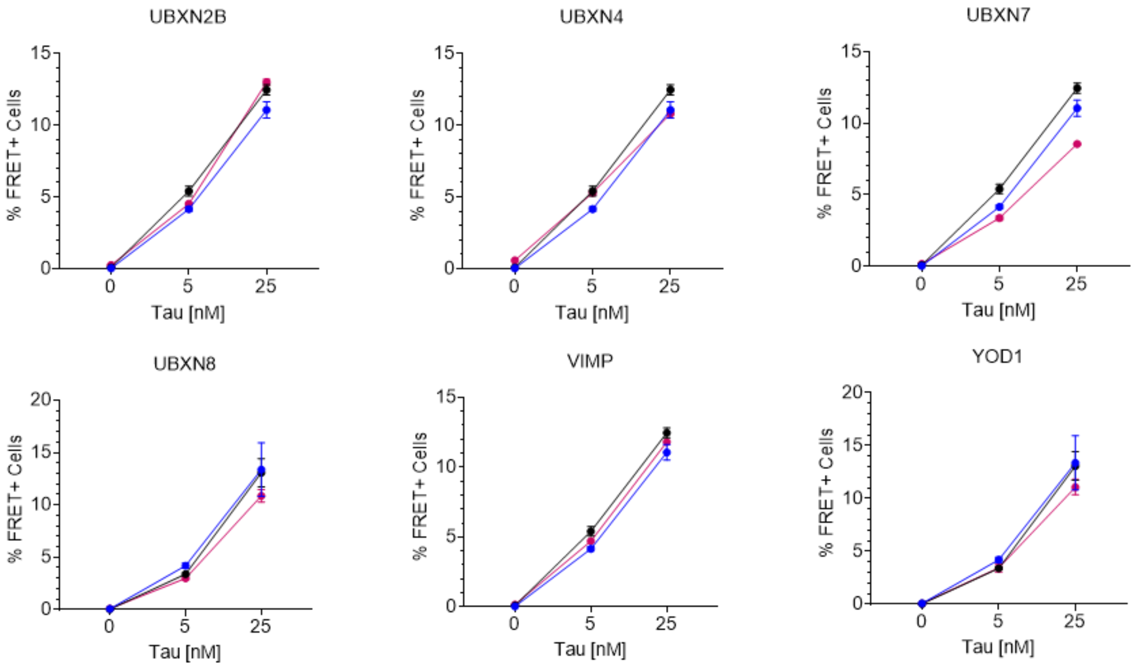

Cofactors tested using siRNA-mediated knockdown:

- Untreated
- Scr
- Cofactor

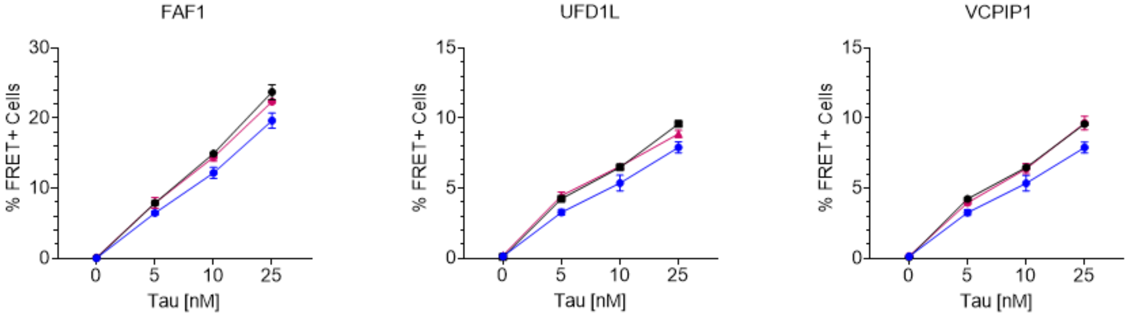

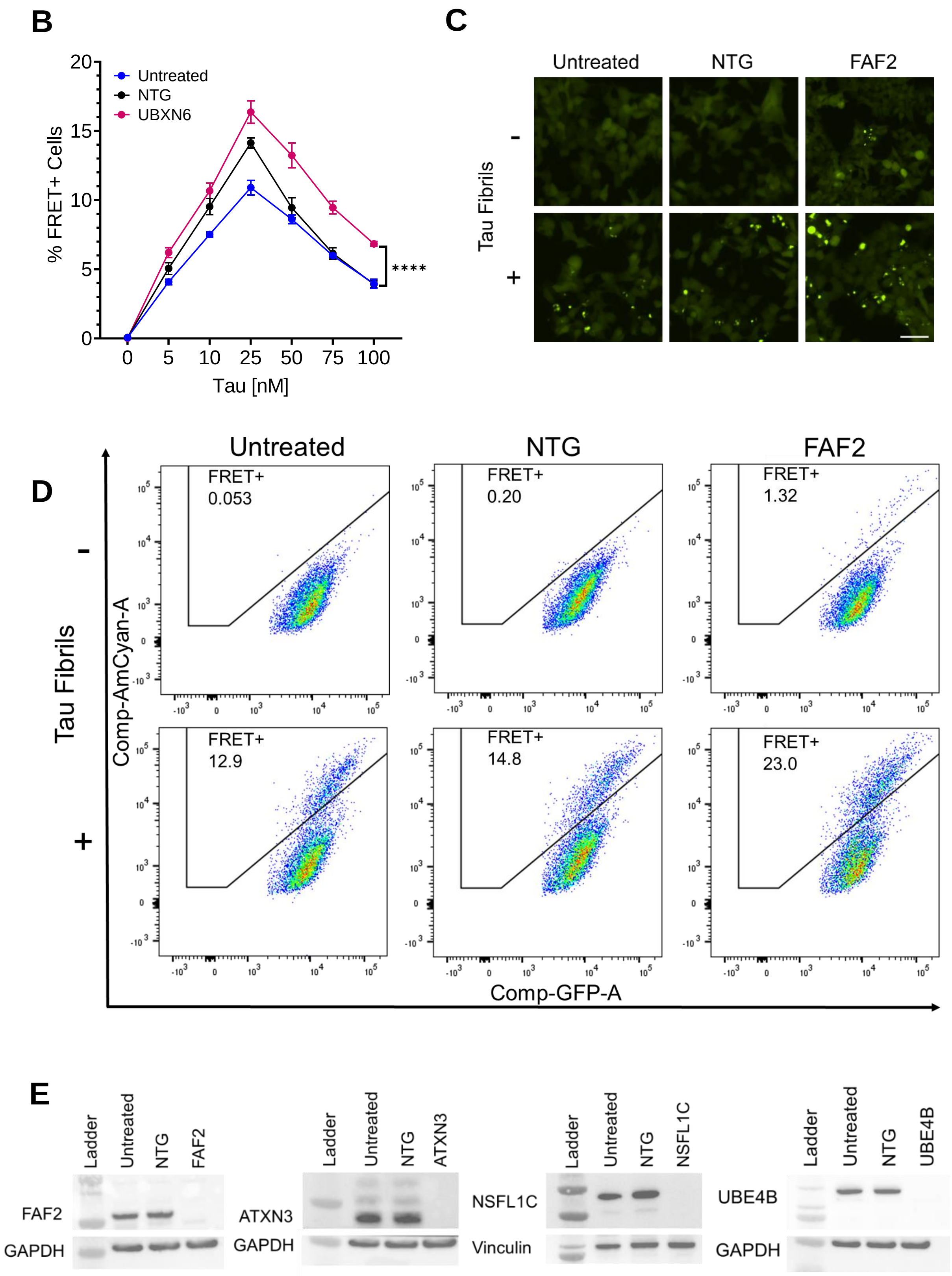
B
% FRET+ Cells
Untreated
NTG
UBXN6
****
Tau [nM]
C
Untreated
NTG
FAF2
Tau Fibrils
-
+
D
Untreated
NTG
FAF2
Tau Fibrils
Comp-AmCyan-A
Comp-GFP-A
FRET+
0.053
FRET+
0.20
FRET+
1.32
FRET+
12.9
FRET+
14.8
FRET+
23.0
E
Ladder
Untreated
NTG
FAF2
GAPDH
ATXN3
NSFL1C
Vinculin
UBE4B

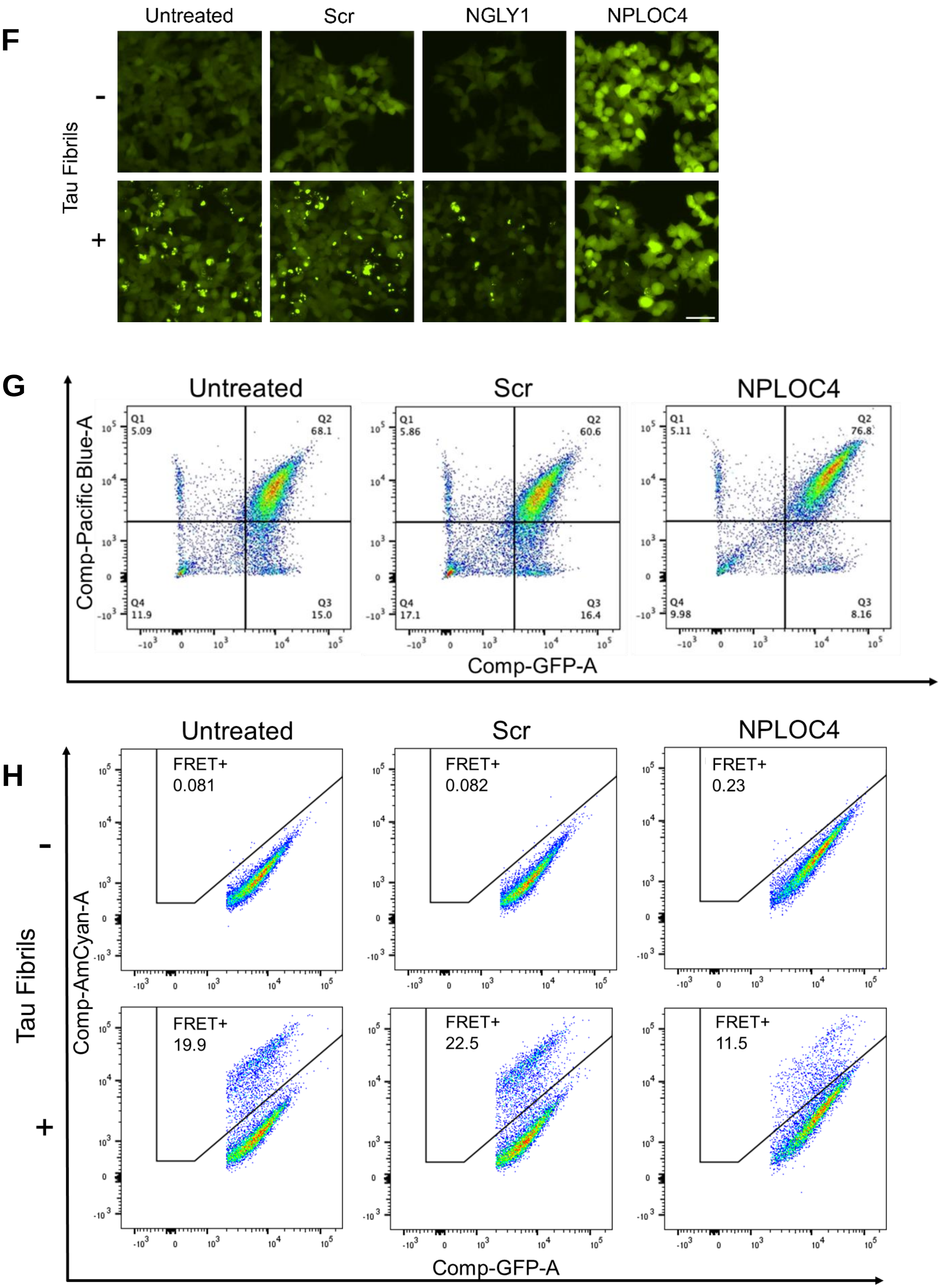
F
Untreated
Scr
NGLY1
NPLOC4
Tau Fibrils
-
+
G
Untreated
Scr
NPLOC4
Comp-Pacific Blue-A
Comp-GFP-A
Q1 5.09
Q2 68.1
Q4 11.9
Q3 15.0
Q1 5.86
Q2 60.6
Q4 17.1
Q3 16.4
Q1 5.11
Q2 76.8
Q4 9.98
Q3 8.16
H
Untreated
Scr
NPLOC4
Tau Fibrils
-
+
Comp-AmCyan-A
Comp-GFP-A
FRET+ 0.081
FRET+ 0.082
FRET+ 0.23
FRET+ 19.9
FRET+ 22.5
FRET+ 11.5

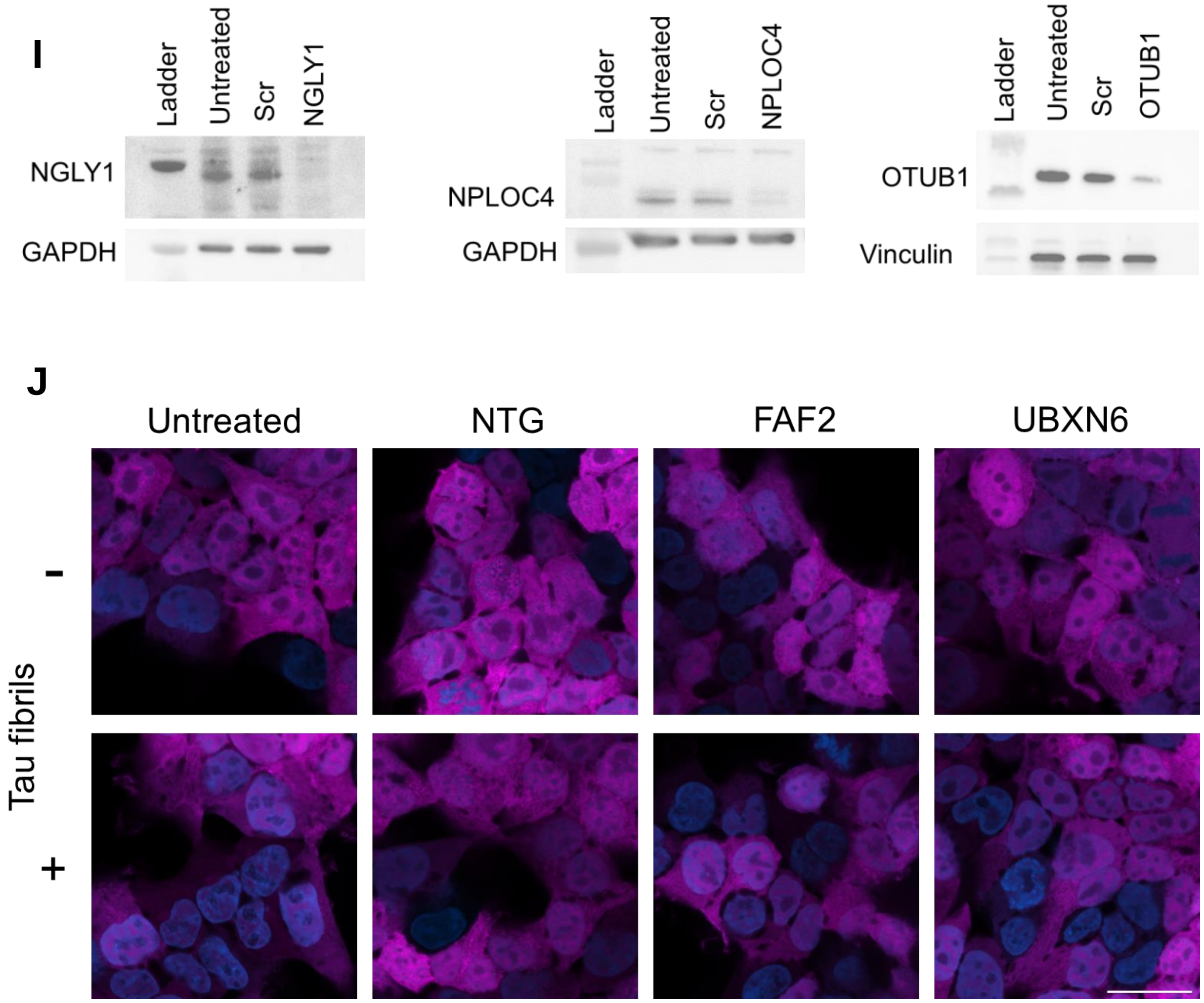
I
Ladder
Untreated
Scr
NGLY1
NGLY1
GAPDH
Ladder
Untreated
Scr
NPLOC4
NPLOC4
GAPDH
Ladder
Untreated
Scr
OTUB1
OTUB1
Vinculin
J
Untreated
NTG
FAF2
UBXN6
Tau fibrils
-
+
